# Boreal forests are heading for an open state

**DOI:** 10.1073/pnas.2404391121

**Published:** 2024-12-30

**Authors:** Ronny Rotbarth, Egbert H. van Nes, Marten Scheffer, Milena Holmgren

**Affiliations:** ^a^Environmental Sciences Department, Wageningen University & Research, Wageningen 6708 PB, The Netherlands

**Keywords:** biome shifts, boreal forests, climate change, ecosystem modelling, resilience

## Abstract

The boreal forest biome is warming four times faster than the global average. Changes so far are moderate, but time lags in responses may transiently maintain forest states which are no longer supported by current environmental conditions. Here, we explore whether tree cover dynamics hint at the state to which the biome may be shifting. Our results suggest that global boreal tree cover is heading for a unimodal state of open forest (30 to 50%), contrasting starkly to the bimodal distribution of dense forests (>60%) and sparse woodlands (5 to 15%) found today. A future open forest in a warmer world would likely increase the risk of forest fires, leading to potentially substantial releases of stored carbon.

## Significance

We apply an innovative modeling approach to project that global boreal forests will undergo a fundamental transition toward an open forest state of 30 to 50% tree cover in the coming decades. We present evidence that such a transition is already underway. The projected shift toward an open forest would alter fire regimes and the carbon storage capability of boreal forests with important implications for global climate regulation. Our approach can be applied to ecosystems beyond boreal forests and is thus a promising addition to the toolkit of ecosystem modeling.

Climate change is expected to alter the distribution, structure, and functioning of global biomes (1). The boreal forest biome lies within a zone of strong warming. As a result, growth conditions for plants and disturbance regimes by fire or insect outbreaks are changing rapidly (2). This may have considerable impacts on the services boreal forests provide for climate regulation, biodiversity conservation, and human livelihoods (3).

The response of boreal forests to changing climatic conditions may occur over decadal to centennial time scales. Such a lag in response is sometimes referred to as “living on borrowed time” (4) or “extinction debt” (5). Lagged shifts in forest states would imply a future redistribution of biodiversity, carbon storage, and timber resources. If these redistributions occur along latitudinal gradients, they may indicate a shift or a contraction of the entire biome (6, 7). As boreal forests constitute one of the largest carbon pools on Earth (8, 9), marked shifts in forest carbon would have direct consequences for climate regulation.

**Fig. 1.**
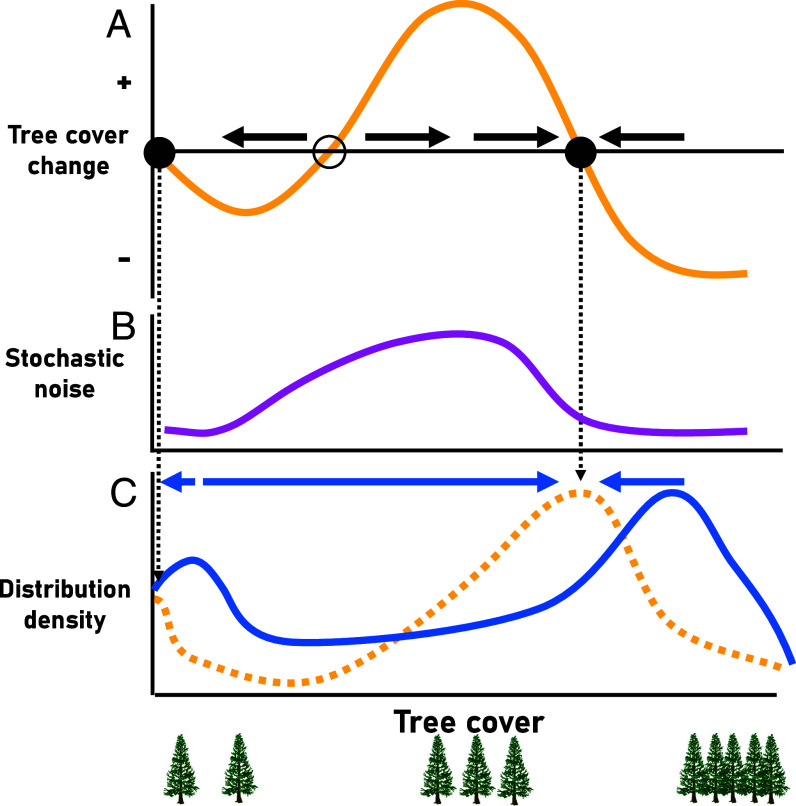
Schematic overview of the detection of disequilibrium in boreal tree cover. Projected future tree cover is a combination of a deterministic change function of tree cover (*A*) and a stochastic noise function around the change (*B*). Circles in A mark tree cover states at which tree cover is in equilibrium. Solid circles are stable equilibria to which tree cover is moving (black arrows). The hollow circle represents an unstable equilibrium. The deterministic and stochastic functions are used to simulate tree cover over time, leading to a probability density distribution of tree cover (yellow dotted line in *C*). Peaks in distributions are expected around the stable equilibria with variation around them based on stochastic noise. This distribution density landscape may be different from current tree cover distribution densities (blue solid line), indicating that the current situation is transient (blue arrows).

A simple measure of forest structure which allows a quantification of forest change over large spatial scales is tree cover. Tree cover often relates to forest biomass carbon and the risk of forest fires (10–12). Here, we analyze dynamics of boreal tree cover (13) over the past twenty years to explore whether tree cover dynamics hint at the state to which the biome may be shifting. This allows a projection of tree cover change over the coming century. We provide an overview of the approach in Fig. 1 (14). In a nutshell, we first assess how tree cover change is deterministically related to actual tree cover (Fig. 1*A*). We then estimate the stochastic noise of tree cover change across the tree cover gradient (Fig. 1*B*). Finally, the resulting deterministic change function and stochastic function allow a projection of the expected long-term distribution of forest states (Fig. 1*C*).

## Results and Discussion

### Recent Tree Cover Distribution and Dynamics.

We extracted tree cover data over the period 2000–2020 from 2,000,000 random sample plots of 6.25 ha in size across the global boreal biome (*Materials and Methods*). We separated our samples into ranges of 0.5 °C mean annual temperature which is one of the strongest drivers of tree growth and cover in boreal forests (10, 15–17). Consequently, it relates strongly to tree cover distributions (SI Appendix, *Supplementary Text* and SI Appendix, Fig. S22). While precipitation plays an impressive role in tree growth, too, it does not determine tree cover modes in the same way as temperature does (SI Appendix, Fig. S22). Hence, we included only temperature in our model.

**Fig. 2.**
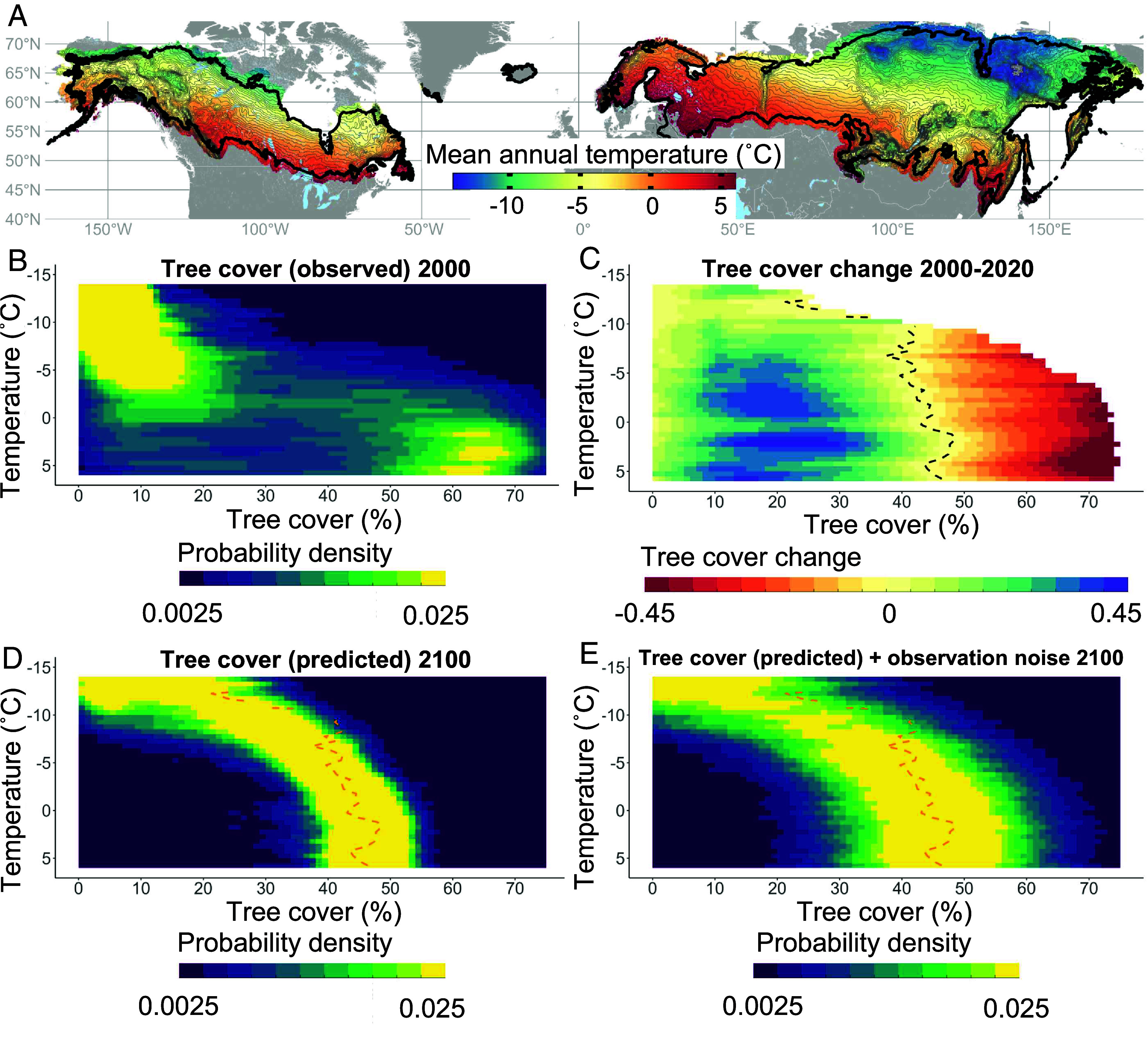
Projected change in boreal tree cover. (*A*) Bins of 0.5 °C of mean annual temperature (thin lines) from 2000–2020 across the boreal biome (thick line) used for tree cover projections. The bins correspond to the y-axis in all panels (reversed y-axis). Temperatures are used as locations within the boreal biome. (*B*) Observed probability densities of tree cover in the year 2000. (*C*) Tree cover change between 2000 and 2020. The dashed line marks zero change. (*D*) Expected probability densities of tree cover in the year 2100. Tree cover was simulated from the initial values in 2,000 using tree cover changes 2000–2020. The dashed line is the line of zero change. (*E*) Expected probability densities of tree cover in the year 2100 including estimated observation noise.

Temperature-specific tree cover distributions for the year 2,000 reveal two dominant modes: one around 5 to 15% tree cover and the other one around 60% tree cover. The height of these two modes differed depending on mean annual temperature (Fig. 2*A*). The low-density mode occurred throughout the temperature gradient but was most dominant in the cold boreal. High-density forests were only present in the warmer half of the boreal biome. Both tree cover modes thus co-occurred across part of the temperature gradient, a pattern that is consistent with the existence of alternative states (10, 18).

Within temperature ranges, we quantified tree cover change in each sample plot using the robust Theil-Sen slope estimation. Specifically, we determined the median difference between all possible pairs of observations in a 21 y period (2000–2020, *Materials and Methods* for details). We then related this change to initial tree cover in 2,000 using generalized additive model (GAM) fits. To estimate process noise and observation noise, we quantified the change in SD in the same data by fitting GAMs (*Materials and Methods* for details).

We observed a persistent tendency for tree cover to decline in warmer forests, but to increase in colder forests (Fig. 2*B*). Warmer boreal sample plots typically correspond to denser forests along the southern edge of the biome and colder sample plots to sparse, interior and northern forests. In practice, our results therefore imply that thinning was common in the (dense) southern forests, while (sparse) interior and northern forests became denser.

### Expected Forest States from Observed Dynamics.

The observed change as a function of temperature and cover (Fig. 2*B*) hints at potential future tree densities. The border between net tree cover losses and gains (dashed line in Fig. 2*B*) marks equilibria to which tree cover would theoretically converge. Following this reasoning, it may already be seen that future forests are projected to converge to intermediate tree cover (40 to 45%), which is somewhat lower in the colder boreal (25 to 30%). This theoretical equilibrium also varied between continents and depended on forest management and the occurrence of fire (SI Appendix, Figs. S1–S6).

For a more realistic prediction, we used the observed tree cover changes in stochastic model simulations of tree cover starting from the initial state in the year 2000 and running to the year 2100. Hereby, we simulate deterministic tree cover change over time in any sample plot based on observed tree cover change and mean annual temperature at the sample location. In each time step, we also add stochasticity (hence the process noise) given the tree cover at that time.

Our simulations confirmed a tendency for aggregation of tree cover around an open forest state (Fig. 2*C*). It should be noted that observed tree cover values have an observation error. We therefore attempted to separate the stochasticity in real tree cover change (“process noise”) from stochasticity that is due to errors in the satellite inferred tree cover (“observation noise”) related to weather conditions and sensor inaccuracies (*Materials and Methods* and SI Appendix, Figs. S7 and S8). Both types of noise can make the identification of equilibria in dynamical systems harder (19–21). However, adding observation noise to the simulations mainly increased the width of the tree cover distributions (30 to 50%, Fig. 2*D*). Those results thus suggest that based on the tendencies of change in the period 2000–2020, the boreal forest will move toward an open state with a tree cover between 30 to 50% decreasing gradually from the South to the North. We should use the term “open forest” carefully though. As each sample plot consists of a 250 × 250 m pixel, we cannot, for instance, distinguish whether an open forest of 50% tree cover contains a homogeneously open forest of 50% or a forest patch of very dense forest covering 50% of the sample plot. We therefore refer to open forest as the forest state on a broader spatial scale without details on the finer characteristics of this state.

### Comparison of Observed and Expected Forest States.

The shift of the boreal biome toward an open state suggests that the current bimodal distribution is unstable and represents a transient situation (Fig. 2 *A* and *D*). In the warmer half of the biome, dense forests are expected to thin (by about 20%). In contrast, sparse forests of the cold boreal would become up to 40% denser. Because dense and sparse forests are generally aligned along a latitudinal gradient from south to north, the expected thinning in the south and densening in the north constitute a climate-driven northward redistribution of tree cover within the boreal biome (6, 7).

The squeezing of high- and low-density forests toward an open forest state may stem from changing temperature conditions reflected in the 2000–2020 patterns of change. Most current dense boreal forests occur at the warm, southern margin of the biome. Here, warming may exceed temperature thresholds faster which could trigger growth reductions in boreal tree species (15, 22). Reduced growth can increase tree mortality risk. Together with the increasing impacts of timber logging, forest fires, and insect outbreaks, growth reductions may thus contribute to forest thinning in warmer boreal forests. Although we tentatively simulated change toward the year 2100, our simulations are based on the 2000–2020 period in which much of the predicted warming has not yet unfolded. In our simulations, we attempted to include possible effects of future warming by updating the temperature-specific tree cover change models based on expected climate model projections of temperature until the year 2100 (*Materials and Methods*). However, we cannot fully account for all future effects of climate change on tree cover changes, rendering our results a likely conservative projection of future change. Especially dry forests (e.g., those north of the Great Plains in North America or in inner Asia) may be lost entirely to alternative vegetation types, such as steppe grasslands or shrublands, under continued warming (23–25).

Projections also remain uncertain at colder parts of the biome where tree growth is typically limited by low temperatures. Warming may lift such limitations, leading to accelerated tree growth and reproduction (15, 17). Forests may also expand into some areas of thawing permafrost that formerly prevented their establishment (26). Such processes may explain the densification we observed. Indeed, evidence of increased shrub and tree growth and of their expansion are already observable in parts of the cold boreal and along the boreal-tundra margin (6, 27–29). However, water cover from permafrost thaw and sudden permafrost collapse can lead to browning and die-off of woody vegetation (30, 31). Limitations related to the accuracy of the remotely sensed tree cover data, especially at the colder margin, could further affect our results on a local scale [*Materials and Methods* and (7, 13)]. It therefore remains unclear to what extent and how fast boreal forests may expand at and beyond their current ranges (7, 26).

**Fig. 3.**
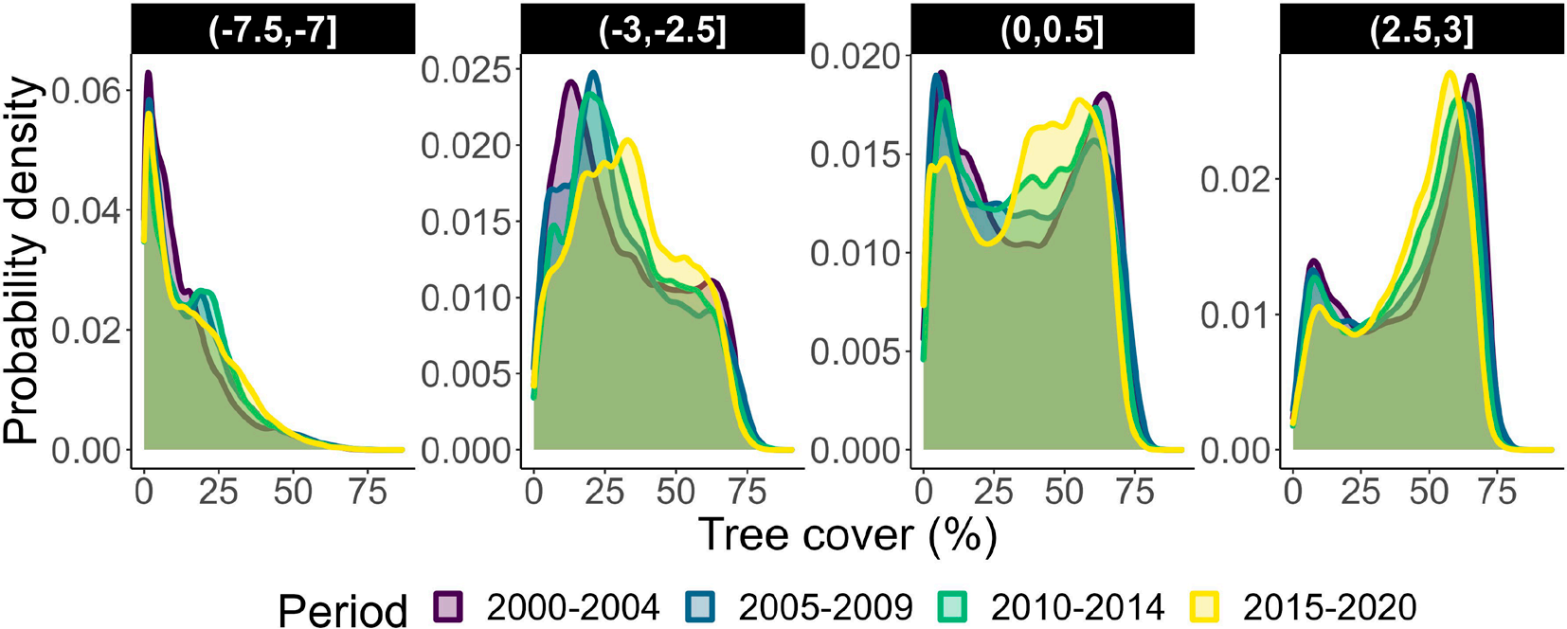
Observed shifts in tree cover distributions 2000–2020. Panels show probability densities of tree cover for four temperature ranges. Ranges are based on mean annual temperatures 2000–2020. Each color represents the densities of all tree cover values during time periods of five to six years between 2000 and 2020.

If climate warming in conjunction with disturbance effects would shift forests to an open state, as our results suggest, we should expect the beginning of such shifts to be already observable over the past decades. Indeed, we found a progressive shift toward the expected open state in tree cover distributions over 5-y periods in most temperature ranges (Fig. 3). These observations are consistent with our model projections over the same period (SI Appendix, Fig. S9). The fact that noticeable systematic tree cover shifts already occurred over the short period of 20 y supports the idea that changes may be relatively fast in some areas which may reach the open state much earlier than 2100.

### Potential Climate Feedbacks.

**Fig. 4.**
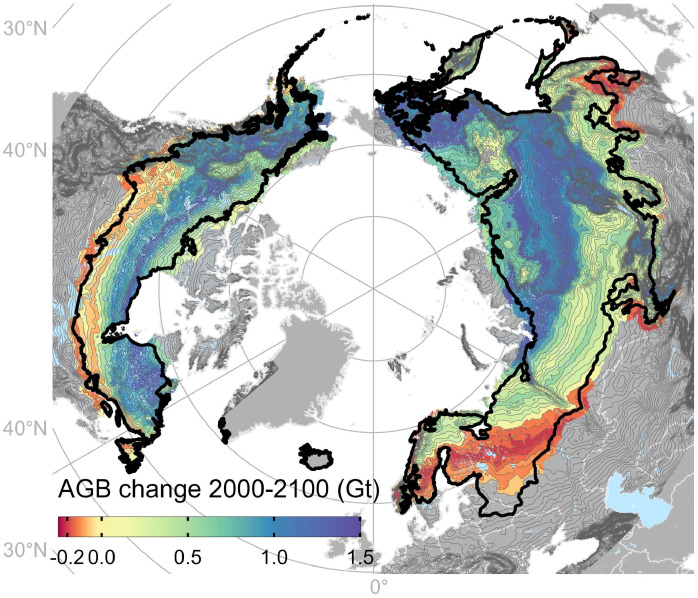
Change in boreal forest aboveground biomass (AGB) between 2000 and 2100. Biomass changes are based on simulated tree cover changes and are total sums of each temperature range (gray contours) over the entire period 2000–2100 (SI Appendix, Fig. S16 for relative biomass changes). Temperature ranges are based on mean annual temperatures 2000–2020. Thick black lines indicate the boreal biome boundary. Areas in gray were not included in analyses.

The projected changes to the boreal forest are massive and may well impact climate through shifts in carbon storage. The redistribution of tree cover we expect indicates that dense forests of high biomass would experience biomass losses, while sparse forests of low biomass would accumulate biomass. To quantify these contrasting shifts, we related tree cover with forest aboveground biomass data. There is a clear relationship between tree cover and biomass (SI Appendix, Fig. S10) which allowed us to estimate how shifts in tree cover would affect carbon storage in future biomass distributions. A shift to an open forest state across most of the current boreal distribution range would imply an estimated net aboveground carbon uptake of 17.7 Gt by 2100 (7.5 Gt in North America and 10.2 Gt in Eurasia, Fig. 4). This constitutes an 11.4% increase compared to current aboveground carbon levels. Our results thus imply that increases associated with the densification of sparse forests could thus exceed the losses incurred by forest thinning in dense forests. Such net biomass gains would be consistent with past decadal biomass changes in boreal forests (32). The latitudinal pattern of biomass carbon change (Fig. 4) also hints at the role of the northern half of the boreal range as possible refugia for the (open) forests in the future (33).

Despite the expected net increase in aboveground biomass carbon, the overall future of boreal forests could well be characterized by a carbon release for three reasons. First, the net increase of aboveground carbon will eventually level off with tree density and when forests reach their northern terrestrial limits at the Arctic Ocean. Second, future forests may be shorter in height and thus store less biomass for any given tree cover density (34). Last, forest expansions in the cold boreal will partly be enabled by a shrinkage in permafrost extent as a result of warming. The thawing of permafrost would release vast amounts of the much larger soil carbon pool (9) and could outweigh the additional aboveground biomass uptake across most of the boreal biome.

A shift of boreal forests to an open forest state would also increase fire risk (SI Appendix, Fig. S15). Low and high-density forests generally burn less than open forests, as they either lack fuel (low density) or do not provide microclimatic conditions for fires to persist (dense forests) (12). This would at least partly explain the dominance of both tree density states across most of the boreal biome (Fig. 2*A*) (10, 18). It is therefore likely that large parts of a future open forest will experience novel fire regimes. Some of these forests, especially in the southern or drier range of the biome, may not be able to recover from severe fires (35, 36), resulting in forest loss. If these losses are extensive enough, they may force a contraction of the boreal biome (7). Future fire regime shifts are challenging to anticipate and may lead to unforeseeable consequences for biome carbon storage and the climate regulation of boreal forests.

In conclusion, our analyses reveal that the boreal forests are in a state of disequilibrium. The observed directions of change are pushing the region toward half-open forest, instead of the two contrasting states that characterize the boreal now. How this massive change might affect the global and regional climate through altered carbon storage and changing disturbance regimes is hard to foresee.

## Materials and Methods

### Study Design.

We defined the global distribution of the boreal forest biome using the boundary delineation in (3). To assess potential tree cover changes in the boreal-tundra and boreal-temperate ecotone transition zones, we created a buffer of 150 km around the north and south boundaries of the boreal forest biome using ArcPro 2.8.3. Within this extended area, we randomly allocated 10,000,000 sample points with a minimum distance of 354 m between points to ensure that each sample would lie within a different pixel of the MODIS tree cover layer (250 m × 250 m) (13). Each sample location refers to pixel in the MODIS dataset and therefore has an area of 250 m × 250 m (6.25 ha) in size. We excluded all points classified as bare ground, crops, oceans, snow/ice, urban, or water on the Global Land Cover Map. From the remaining points, we randomly selected 2,000,000 points which were equally allocated to the North American and Eurasian boreal forests (i.e., 1,000,000 points each). The sample points cover an area of 12,500,000 ha based on the MODIS pixel size. We then extracted data from all datasets (SI Appendix, Table S1) for each of the sample points. We tested the representativeness of the samples by comparing tree cover distributions, fire proportional areas, and forest management classes between sample points and the entire biome (SI Appendix, Fig. S17; Table S2). All comparisons highlighted a strong representativeness of sample points regarding fire disturbance, forest management, and tree cover distributions.

### Datasets and Preprocessing.

The datasets used in our study and steps involved in preprocessing are shown in SI Appendix, Table S1. The central dataset of MODIS tree cover is a widely used and generally accurate estimation of global vegetation cover (including tree cover) (37, 38). It has been used in a wide range of research. Along the cold margin of boreal forests where tree cover is low, the dataset may be less accurate because small trees in this region are masked due to a height threshold of 5 m (7, 37–39). It is for these potential inaccuracies that we considered observation noise in our model projections based on observable data (see details below). We further assess tree cover projections on a global scale which are less affected by inaccuracies due to local or regional circumstances.

The dataset on aboveground biomass is likewise a commonly used dataset which is accurate on a regional to global scale. It performs particularly well at the low to intermediate range of biomass which is the range dominant in boreal forests (40).

### Modeling Approach.

Stochastic noise plays an important role in modeling ecological systems. Without variations around the modeled dynamics, a system will converge toward stable states. In the case of boreal forests, we would expect modeled tree cover values to all move toward one or more individual tree cover states. As this scenario is unrealistic, we included noise in our model to account for the natural variation in tree cover changes. Such variations may be driven by differences in soil properties, climatic conditions, disturbances, topographic location, etc.

The strength of noise is crucial, as it determines not only the spread of the system around stable states, but it may even prevent the system from converging toward one or more states. We assumed that the variation in the MODIS tree cover time series is composed of two parts: process noise and observation noise. Process noise is the natural variation of processes like growth and perturbations that are inherent to the system. Observation noise is caused by inaccuracies in measurements, e.g., directly by the observer or by inaccuracies in the used measurement devices. The latter is particularly relevant for remote sensing data, as sensor sensitivities or atmospheric interference can cause variations in the data which are unrelated to the measured system itself.

Based on these considerations, we used a dynamic stochastic tree cover model to project tree cover change into the future. Our tree cover model describes tree cover change as a stochastic differential equation, more specifically the Langevin equation (41).

This model consists of a deterministic f[X(t)] and a stochastic part g[X(t)] that together describe the stochastic dynamics of the system. Both model parts are unknown functions of tree cover X(t):

[1] $$dX_{t}=f\left(X_{t}\right)dt+g\left(X_{t}\right)dW_{t},$$

In this function, $dW_{t}$ is the normally distributed increment of the stochastic Wiener noise term. We use the Euler–Maruyama scheme to solve this stochastic differential equation:

[2] $$X_{t+\Delta t}=X_{t}+\Delta t\;f\left(X_{t}\right)+\sqrt{\Delta t}\;g\left(X_{t}\right)\xi_{t+\Delta t},$$

where X_t_ is the tree cover at time t, Δt is the time step for simulation (we used 0.1 y), f($X_{t}$) and g($X_{t}$) are unknown functions that are estimated from the data, and $\xi_{t+\Delta t}$ is a white noise process, consisting of independently drawn numbers from a normal distribution with zero mean and a SD of one. Additionally, we considered that there is uncertainty in the determination of the tree cover ($X_{t}$). Therefore, we assume there is uncorrelated observation noise, that could be dependent on the tree cover [$h\left(X_{t}\right)$], and is superimposed on the generated stochastic time series:

[3] $${\hat{X}}_{t}=X_{t}+h\left(X_{t}\right)B_{t},$$

where ${\hat{X}}_{t}$ is the resulting time series including observation noise; $X_{t}$ is the generated time series of tree cover using Eq. **2**, and $B_{t}$ is a stochastic process derived from a standard normal distribution with zero mean and a SD of 1.

### Data Selection for Model Fitting and Simulation.

Our model describes tree growth assuming fixed environmental conditions. Therefore, we fitted the model functions independently for different sets of data points, that were selected to keep the environmental conditions as similar as possible. Specifically, we selected datasets based on four main drivers: 1) mean annual temperature, 2) geographic region, 3) fire occurrences, and 4) forest management.

Temperature is one of the main drivers of tree growth in high-latitude systems and has been linked to observable boreal forest states (10, 18, 42). We classified sample points into temperature ranges of 0.5 °C between −14 °C and 6 °C. This temperature span constitutes the most frequently observed mean annual temperatures within our study area (SI Appendix, Fig. S18). Additionally, boreal forests in Eurasia and North America differ considerably in climatic conditions, disturbance regimes, and species compositions. We therefore separated sample points by these two geographic regions for model fitting and simulation. In a similar manner, we selected sample points based on recent fire occurrence from the MODIS Burned Area dataset (SI Appendix, Table S1). We hereby selected only fires that occurred within our study period 2000–2020. We also selected by whether sample points lied in managed or unmanaged forests based on the forest management map and classifications in SI Appendix, Table S1.

It is important to note that we always separated sample points in temperature bins, irrespective of any other selection criteria. For example, when we separated by geographic region, we additionally separated by temperature within North America and Eurasia.

### Calculation of Tree Cover Change.

We used observed tree cover change to estimate the deterministic part of our model $f\left(X_{t}\right)$. To reduce the effect of observation noise, we determined tree cover change by analyzing the trend in tree cover in the period between 2000 and 2020. Within each sample plot, we quantified tree cover change as the median annual difference in tree cover using the Theil-Sen-slope estimation. This method first calculates the slope between each point in the time series with every other point. In a second step, the overall slope is calculated by taking the median of all these slopes. The result is an annual rate of absolute tree cover change in % year^−1^. This method is commonly used for robust trend estimations in time series with high observation noise which may be present in remote sensing time series. We used the “zyp” package version 0.10-1.1 in R to apply the Theil-Sen-slope estimation (43).

### Fitting the Deterministic Model Part ***f***(***X_t_***).

To assess tree cover change along the tree cover spectrum, we related initial tree cover in the year 2000 with the tree cover changes determined for all selected sample points using the method of Rotbarth et al. (2023) (7). Contrary to a parametric approach using defined function families, this method fits the relationship through nonparametric GAM. GAMs can deal with nonlinear relationships between variables. In essence, GAMs split the observed range of variable x (in our case initial tree cover) into smaller sections. Within each section, x (initial tree cover) and y (tree cover change) are then fitted with polynomial functions (we used a cubic regression smoother). Each of these separate function fits is then combined to form the overall fit of the GAM. This predictive model formed the deterministic part in the tree cover model ($f\left(X_{t}\right)$ in Eq. **2**). GAMs also formed the basis for fitting $g\left(X_{t}\right)$ and $h\left(X_{t}\right)$. We fitted GAMs using the “mgcv” package version 1.8-10 (44).

### Fitting the Stochastic Model Part: Process Noise ***g***(***X_t_***) and Observation Noise ***h***(***X_t_***).

A challenge in accounting for process and observation noise in real data is the separation between the types of noise. Current approaches so far have treated noise as the sum of process and observation noise (14). Here, we followed a simple approach for noise separation. We first identified all sample points with the same tree cover in any given year between 2000 and 2020. For each group of points with the same tree cover (groups of 1%), we calculated the SD of tree cover in the following years Δt, with Δt ranging from 1 to 10 y. As SD are thus measured over time, we would expect variations to increase because the initial tree cover values will develop in a divergent manner, i.e., some may decrease while others will increase. According to SI Appendix, Eq. **S2**, for small time steps, we would expect the process noise to increase approximately proportional to the square root of the time step, while observation noise should be independent of the time step. The expected relationship between the increase in SD [σ(Δt)] and time intervals (Δt) should thus be

[4] $$\sigma (\Delta t)=a+b\sqrt{\Delta t},$$

The slope b represents the process noise of the system, as it determines how fast the variation increases over time. The intercept a represents the observation noise that is independent of the time step.

We tested the above theoretical relationship by performing linear regressions using SI Appendix, Eq. **S4**. We found relationships between SD and time steps for most tree cover values (SI Appendix, Fig. S19). We extracted the slopes b as process noise and the intercepts a as observation noise for each group of sample points with the same tree cover. This allowed us to plot the relationship between tree cover and process noise, respectively observation noise. These relationships form the basis for model fitting $g\left(X_{t}\right)$ and $h\left(X_{t}\right)$.

For process noise, we used GAMs to fit the relationship $g\left[X\left(t\right)\right]$ between process noise (i.e., b) and tree cover analogue to fitting $f\left[X\left(t\right)\right]$. Process noise may vary with temperature. We tested this by fitting separate functions for different temperature ranges (40 bins of 0.5 °C). The overall relationship between process noise and tree cover turned out to be similar across temperatures (SI Appendix, Fig. S7) and the expected tree cover did not differ considerably (SI Appendix, Fig. S20). For simplicity, we used one temperature-independent noise model $g\left[X\left(t\right)\right]$ in the tree cover model, yet still fitted separate noise models for the other selection criteria of geographic region, fire, and forest management.

Similar to process noise, we fitted GAMs for observation noise a in SI Appendix, Eq. **S4** to describe the relationship between tree cover and observation noise $h\left(X_{t}\right)$ (SI Appendix, Fig. S8). Like process noise, observation noise did not seem to be dependent on temperature. Therefore, we also used one relationship between tree cover and observation noise. We added observation noise to the final tree cover output of the simulations (see also SI Appendix, Eq. **S3**) to compare the model output directly with current tree cover observations from MODIS which also include observation noise.

### Simulations.

We used Eq. **2** based on the fitted deterministic part $f{(X}_{t})$ and the stochastic part $g{(X}_{t})$ to project tree cover into the future. The simulations were performed independently for each subset based on sample criteria described in “Data selection for model fitting and simulation.”

We randomly selected 80,000 samples from our selected points, whereby we ensured that these were equally distributed within the 40 temperature bins of 0.5 °C (i.e., 2,000 samples per temperature bin). We then initialized the simulation with the tree cover values from the year 2000 for each of the selected samples.

Using the fitted Eq. **2**, we simulated tree cover for each selected sample point from t = 0 (i.e., year 2000) to t = 990 (i.e., year 2100). As we fitted the model function $f{(X}_{t})$ for each temperature bin independently, we simulated tree cover using the respective model $f{(X}_{t})$ for the associated temperature bin of each sample point. Note that $g{(X}_{t})$ was not fitted for each temperature, as described under “Data selection for model fitting.”

We finally added observation noise to the simulated tree cover (Eq. **3**). We chose the specific value of observation noise based on simulated tree cover and added this noise based on Eq. **3**.

### Incorporating Climate Warming.

Our simulations described above assume that mean annual temperatures in each temperature bin remain constant until the year 2100. To project the effect of future warming, we made the following adjustments to the simulations described above.

We derived predicted mean annual temperatures for the years 2020–2100 from the EC-Earth3-Veg-LR model for scenario SSP3-7.0 (SI Appendix, Table S1) for each of the 80,000 samples in our simulations. Between 2000 and 2020 we used the mean annual temperature in this period. After that, the model checks whether predicted temperatures for each simulated year and sample lie in a different temperature range than the initial range. The simulation is then updated by using the respective tree cover change model of the new temperature range. This allows tree cover to be modeled beyond the initially observed tree cover range in colder regions. However, updating tree cover dynamics was not possible in regions that are expected to experience warming beyond the currently observed warmest temperatures. In these cases, the model of the warmest temperature range was used.

### Testing Fitted Models.

We tested our fitting method using model-generated data. Specifically, we tried to recover the model equations using simulated datasets. We tested three datasets generated with different stochastic differential equations: 1) logistic growth with additive noise, 2) Allee effect model with additive noise, and 3) strong Allee effect with additive noise. We chose these models because they are well-known population models that resemble the observed tree cover functions. The three models and the test results are shown in supplementary information.

### Biomass Changes.

We found strong trends between forest aboveground biomass and tree cover for the years 2010 and 2018. We identified the model with the best fit based on lowest AIC for North America and Eurasia separately. The best models were exponential models for the year 2018 (SI Appendix, Table S3). We used these models to calculate total biomass in the year 2100 based on tree cover simulations in each temperature range. We then quantified differences between 2000 and 2100 of each simulated point. We then calculated relative biomass change per temperature range and continent (SI Appendix, Fig. S16). We quantified the total area of each temperature range and converted relative biomass change to total biomass change per temperature range which we presented in the main text.

## Supplementary Material

Appendix 01 (PDF)

Code S01 (R)

## Data Availability

The source code for data modeling is availableas supporting information. the data tables, including information on all variables used in the modelling approach, are available on zenodo, https://doi.org/10.5281/zenodo.14104854 (45). Tree cover data extracted from MODIS VCF, Collection 6 and the MODIS Burned Area dataset are available via the Application for Extracting and Exploring Analysis Ready Samples (AρρEEARS). ERA5 climatic data on surface temperatures are available on the Copernicus website, https://doi.org/10.24381/cds.f17050d7 (46). Projected temperatures from the EC-Earth-Veg_LR model are available on the Copernicus website, https://doi.org/10.24381/cds.c866074c (47). The global land cover map is available on Zenodo, https://doi.org/10.5281/zenodo.3939038 (48). The map on forest management classes is available on Zenodo, https://doi.org/10.5281/zenodo.5879022 (49). The forest aboveground biomass maps are available through the Centre of Environmental Data Analysis, https://doi.org/10.5285/5f331c418e9f4935b8eb1b836f8a91b8 (50). The boreal forest boundary data to delineate our study region is currently being transferred to a publicly accessible repository. Until this transfer is complete, the dataset is available through Dominique Boucher, dominique.boucher@NRCan-RNCan.gc.ca.
