# Supplementary material for: Boreal forests are heading for an open state
Source: Proc Natl Acad Sci U S A. 2024 Dec 30;122(2):e2404391121. doi: 10.1073/pnas.2404391121 (PMC11745404; doi:10.1073/pnas.2404391121)
Supplement: Supplementary file 1 — Appendix 01 (PDF) [file pnas.2404391121.sapp.pdf]

PNAS

# Supporting Information for

## Boreal forests are heading for an open state

Ronny Rotbarth1*, Egbert H. van Nes1, Marten Scheffer1, Milena Holmgren1

1 Environmental Sciences Department, Wageningen University & Research; Droevendaalsesteeg 3 a, 6708 PB, Wageningen, The Netherlands.

*Ronny Robarth

Email: ronny.rotbarth@wur.nl

**This file includes:**

Supporting text

Figures S1 to S22

Tables S1 to S3

SI References

**Other supporting materials for this manuscript include the following:**

Software S1

## Supporting Information Text

### Testing fitted models

The following simple quadratic or cubic model equations were used to describe the Allee effect for model testing(1):

Logistic growth:

$$dX = r\,X\,\left(1 - \frac{X}{K}\right)dt + \sigma\sqrt{(X(100 - X)}\,dW_t \qquad \text{(S1)}$$

Allee effect:

$$dX = r\,X\,\left(1 - \frac{X}{K}\right)\left(\frac{X}{C} - 1\right)dt + \sigma\sqrt{(X(100 - X)}\,dW_t \qquad \text{(S2)}$$

Where r is the growth rate (r = 0.01 $year^{-1}$), K is the carrying capacity (we used K = 80%) and C is the unstable equilibrium which is the tree cover needed for positive growth (we tested both C = 10 and C = 30), $\sigma$ is the level of process noise ($\sigma$ = 0.04) and dW/dt is the increment of the Wiener process. The added stochastic noise decreases towards 0 when tree cover moves to 0% or to 100%. We used the Euler-Maruyama method (time step = 0.001 years) to generate 10,000 time series of 21 years. After the simulation, we added random observation noise to the generated time series (normal distribution with zero mean and standard deviation of 1). We used the method described above to fit the deterministic and stochastic parts of the models and compared these fitted model parts with the chosen Allee effect and logistic growth models.

### Deterministic model test

We showed that our approach was able to recover the deterministic model equations, in particular the two stable equilibria at 0% and 80% tree cover were well predicted in all model types (Fig. S11). However, the model failed to reproduce the Allee effect when this effect was small (Fig. S11A) but recovered the Allee effect well when it was bigger (Fig. S11B). Our model was also less accurate in predicting the stable equilibrium at 0% tree cover on the logistic growth model, although overall fit was good.

### Stochastic model test

We performed model fitting with combinations of different process noise (0, 0.02, 0.04) and different observation noise (0, 1, 2 and 3). Both process and observation noise were generally well recovered (Fig. S12 and Fig. S13). Especially the prediction of observation noise was very satisfactory for most combinations (Fig. S13). However, for larger observation noise, our method increasingly underestimates process noise (Fig. S12H, I, K, L). Despite this underestimation, our model performs very well in recovering the general relationship between process noise and tree cover. This means that the small inaccuracies in process noise estimation for large observation noise remains constant across the tree cover spectrum.

In essence, testing the stochastic part of our method showed that in the presence of strong observation noise, process noise may be underestimated. Therefore, we tested the sensitivity of our future projections for process and observation noise below.

### Sensitivity analysis of noise types

To test whether larger process and observation noise influence the outcome of our future projections, we performed simulations under different combinations of process and observation noise. We therefore do not fit the stochastic part of our model to observed data (i.e. $g(X_t)$ in Equation 2 and $h(X_t)$ in Equation 3) but assume different levels of noise (process noise = {0.58, 1, 2, 3}, observation noise = {0, 1, 3, 8.7}). The values 0.58 and 8.7 for process and observation noise are the median of the observed noise. We then repeated the simulation steps described above.

We show that neither combination changes the projected location of modes but merely increases the width of the distribution (Fig. S14). The projected open tree cover state across most temperature ranges remains unchanged irrespective of process or observation noise.

### Test summary

Based on our analyses, we conclude that our method can robustly detect possible forest states based on past tree cover change. However, weak Allee effects may be missed by our model fit, combined with underestimated stochastic noise under some conditions. Whether such Allee effects are present in real data in the first place, remains challenging to detect.

### Test of alternative modelling approach

To test whether the model projections are not a result of the fitting procedure, we applied an alternative approach. We used the same tree cover model in Equation 2 but did not fit the deterministic and stochastic parts via GAMs but quantified tree cover change and noise within individual bins of 1% tree cover. Hereby, tree cover change is the mean trend of 2000-2020 within each bin. Process noise and observation were quantified using Equation 4 for each of the bins. For each tree cover bin, we recorded the slope $b$ (process noise) and intercept $a$ (observation noise). During model simulations, we associated the tree cover bin with the tree cover of each sample and time step and then added the model components of tree cover change and noise to the model using the respective bin.

Model projections of the binned approach were very similar to the ones produced by the approach using GAMs (Fig. S21). The open forest state was likewise projected as a future trajectory. The results are therefore robust against the fitting method.

### Influence of precipitation on tree cover distribution

We included mean annual temperature as the main driver of tree cover distribution in our model. However, precipitation plays another significant role in plant growth and vegetation. In the boreal biome, precipitation in combination with temperature delineates the distinct southern biome margin towards steppes and grasslands in central North America and inner Asia.

For our objective, it is important to determine whether precipitation relates to tree cover distributions to such an extent that an inclusion in our model would be justified. We therefore explored how tree cover distributions change within the precipitation-temperature spectrum across our 2,000,000 samples across the boreal biome. We show the results in Fig. S22. Temperature exerts a much stronger control over shifting tree cover distributions than precipitation (in particular regarding the different tree cover modes/peaks). For example, moving from cold to warmer regions within the

highest precipitation bin (>794mm), shows an emerging tree cover mode around 0-10%, followed by the addition of another mode around 20% at intermediate temperatures. This second mode subsequently shifts toward higher tree cover in the warmest temperature bins. Contrarily, moving along the precipitation gradient from dry to wet within the same temperature bin (e.g. -6 to -4 ˚C) lacks a marked shift in the two existing tree cover modes (around 0-10% and 20%). For the purpose of our model, which aims to project tree cover distributions and modes, the addition of precipitation in the model would therefore unlikely change the results and only increase the complexity, thus reducing interpretability.

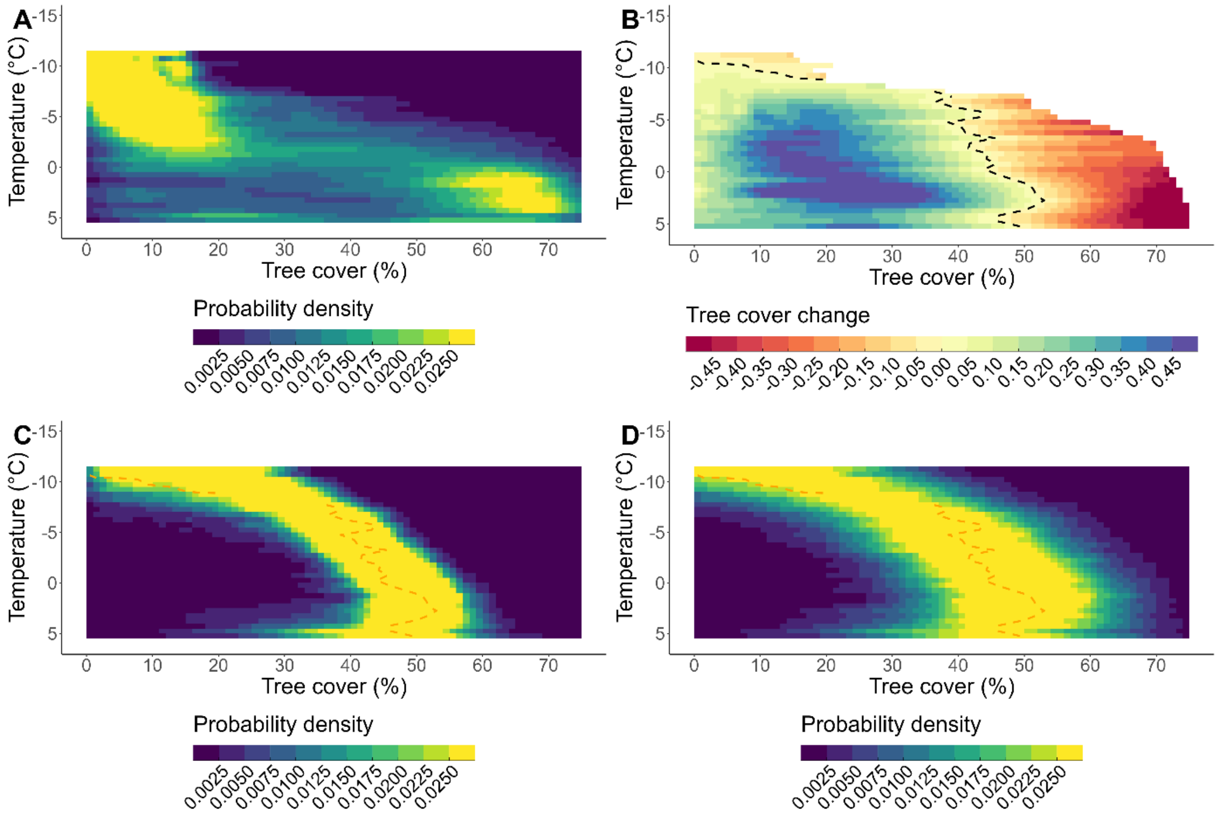


**Fig. S1. Tree cover distribution and change in North American boreal forests.** Each panel represents distributions or change within a tree cover-temperature space. Tree cover is shown in ranges of 1%. Temperatures are mean annual temperatures 2000-2020 and are shown in ranges of 0.5°C. **A** Observed probability densities of tree cover in the year 2000. **B** Tree cover change between 2000 and 2020. The dashed line marks zero change and thus indicates potential tree cover states. **C** Expected probability densities of tree cover in the year 2100. Tree cover was simulated from the initial values in 2000 using tree cover changes 2000-2020. The dashed line is the line of zero change around which tree cover distributions are expected. **D** Expected probability densities of tree cover in the year 2100 including observation noise.

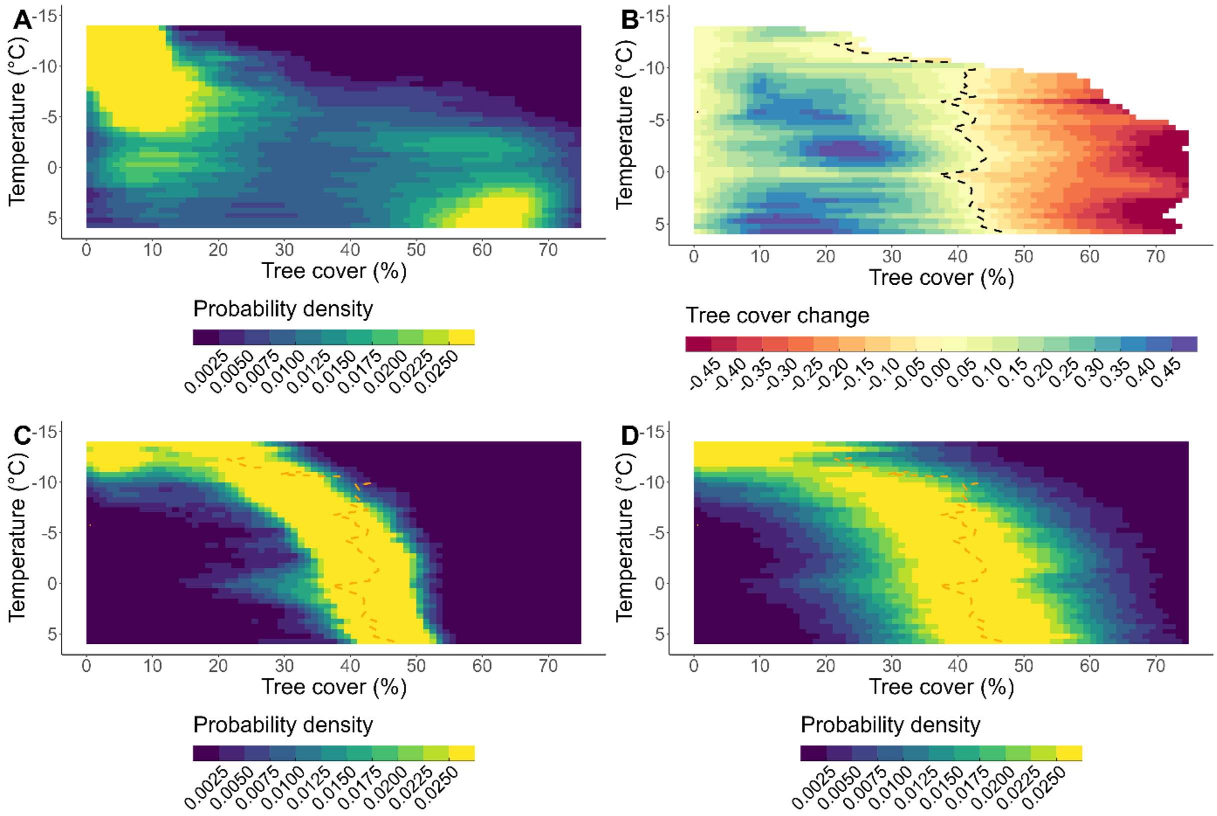


**Fig. S2. Tree cover distribution and change in Eurasian boreal forests.** Each panel represents distributions or change within a tree cover-temperature space. Tree cover is shown in ranges of 1%. Temperatures are mean annual temperatures 2000-2020 and are shown in ranges of 0.5°C. **A** Observed probability densities of tree cover in the year 2000. **B** Tree cover change between 2000 and 2020. The dashed line marks zero change and thus indicates potential tree cover states. **C** Expected probability densities of tree cover in the year 2100. Tree cover was simulated from the initial values in 2000 using tree cover changes 2000-2020. The dashed line is the line of zero change around which tree cover distributions are expected. **D** Expected probability densities of tree cover in the year 2100 including observation noise.

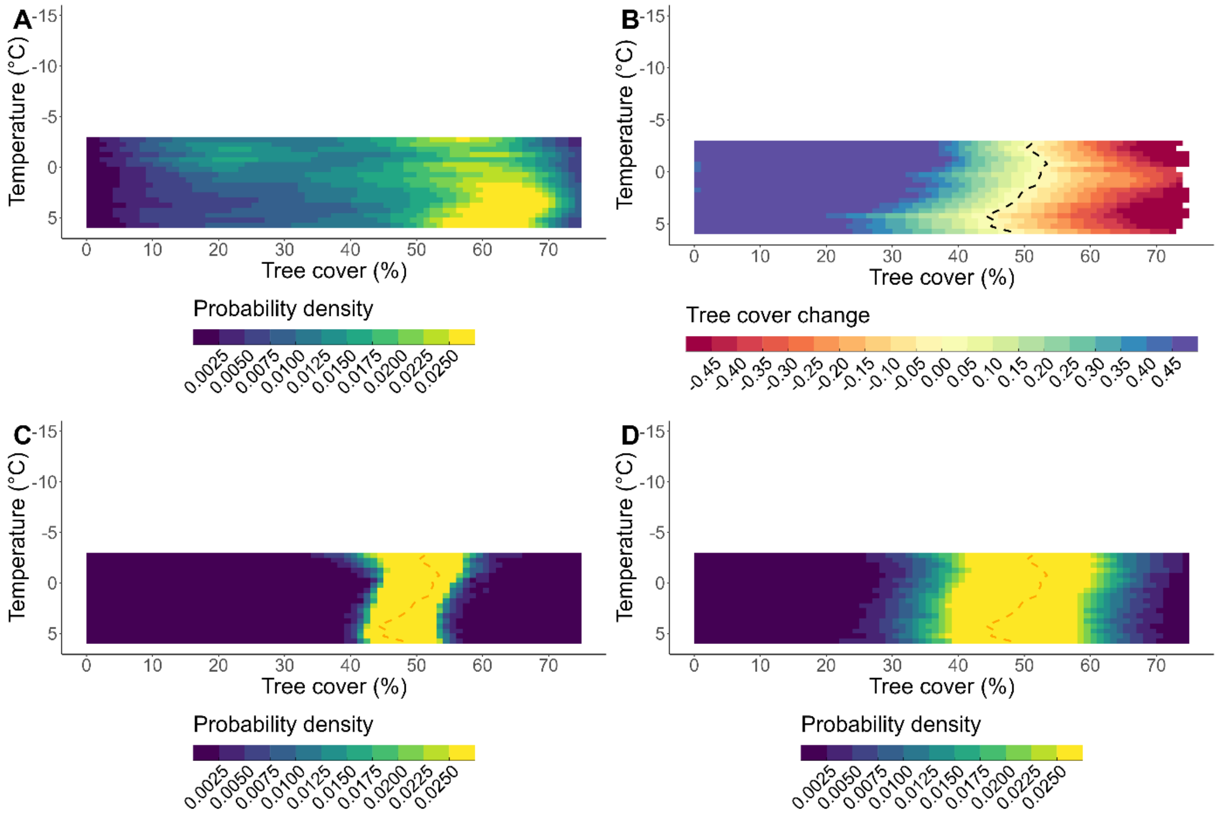


**Fig. S3. Tree cover distribution and change of global boreal forests under forest management.** Forests with any sign of management activity, such as logging or replanting were considered. Each panel represents distributions or change within a tree cover-temperature space. Tree cover is shown in ranges of 1%. Temperatures are mean annual temperatures 2000-2020 and are shown in ranges of 0.5°C. **A** Observed probability densities of tree cover in the year 2000. **B** Tree cover change between 2000 and 2020. The dashed line marks zero change and thus indicates potential tree cover states. **C** Expected probability densities of tree cover in the year 2100. Tree cover was simulated from the initial values in 2000 using tree cover changes 2000-2020. The dashed line is the line of zero change around which tree cover distributions are expected. **D** Expected probability densities of tree cover in the year 2100 including observation noise.

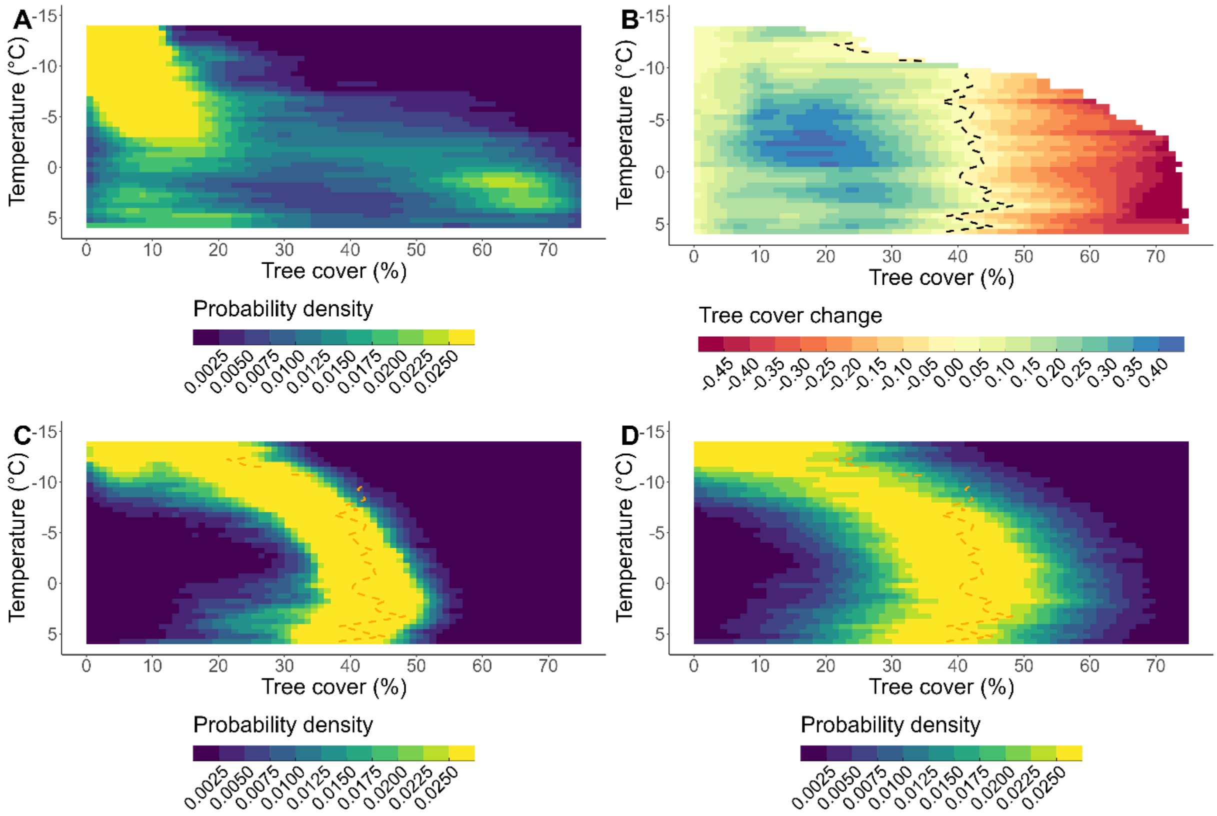


**Fig. S4. Tree cover distribution and change of unmanaged global boreal forests.** Only forests without any sign of human intervention were considered. Each panel represents distributions or change within a tree cover-temperature space. Tree cover is shown in ranges of 1%. Temperatures are mean annual temperatures 2000-2020 and are shown in ranges of 0.5°C. **A** Observed probability densities of tree cover in the year 2000. **B** Tree cover change between 2000 and 2020. The dashed line marks zero change and thus indicates potential tree cover states. **C** Expected probability densities of tree cover in the year 2100. Tree cover was simulated from the initial values in 2000 using tree cover changes 2000-2020. The dashed line is the line of zero change around which tree cover distributions are expected. **D** Expected probability densities of tree cover in the year 2100 including observation noise.

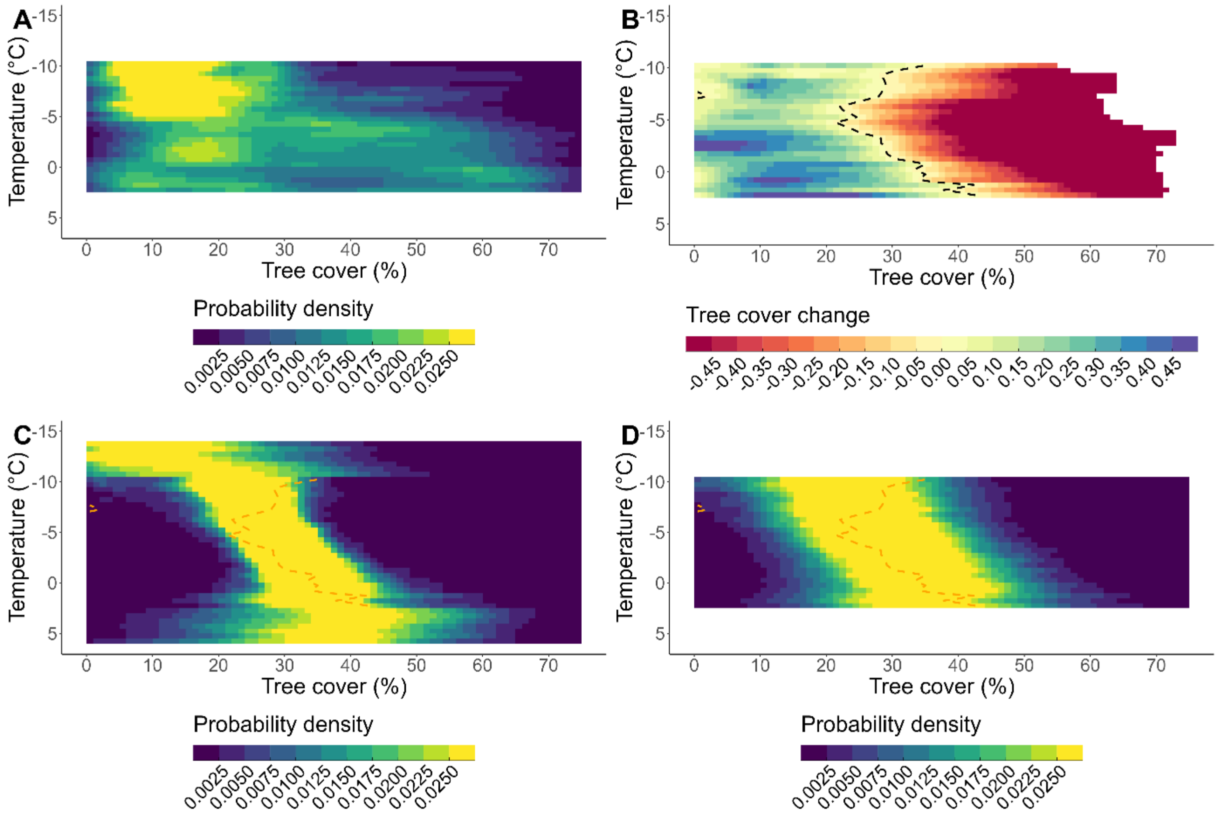


**Fig. S5. Tree cover distribution and change of burnt global boreal forests.** Only forests that burned between 2000-2020 were considered. Each panel represents distributions or change within a tree cover-temperature space. Tree cover is shown in ranges of 1%. Temperatures are mean annual temperatures 2000-2020 and are shown in ranges of 0.5°C. **A** Observed probability densities of tree cover in the year 2000. **B** Tree cover change between 2000 and 2020. The dashed line marks zero change and thus indicates potential tree cover states. **C** Expected probability densities of tree cover in the year 2100. Tree cover was simulated from the initial values in 2000 using tree cover changes 2000-2020. The dashed line is the line of zero change around which tree cover distributions are expected. **D** Expected probability densities of tree cover in the year 2100 including observation noise.

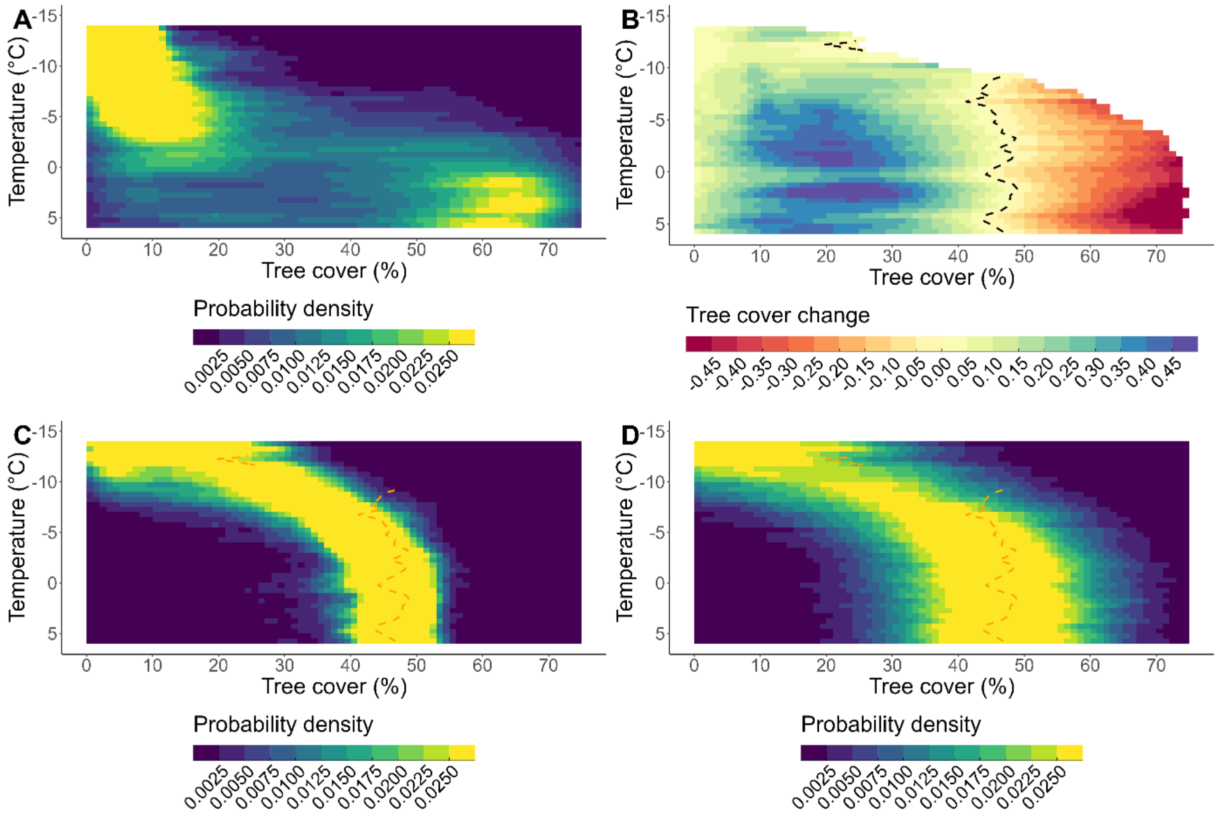


**Fig. S6. Tree cover distribution and change of unburnt global boreal forests.** Only forests that were unburnt between 2000-2020 were considered. Each panel represents distributions or change within a tree cover-temperature space. Tree cover is shown in ranges of 1%. Temperatures are mean annual temperatures 2000-2020 and are shown in ranges of 0.5°C. **A** Observed probability densities of tree cover in the year 2000. **B** Tree cover change between 2000 and 2020. The dashed line marks zero change and thus indicates potential tree cover states. **C** Expected probability densities of tree cover in the year 2100. Tree cover was simulated from the initial values in 2000 using tree cover changes 2000-2020. The dashed line is the line of zero change around which tree cover distributions are expected. **D** Expected probability densities of tree cover in the year 2100 including observation noise.

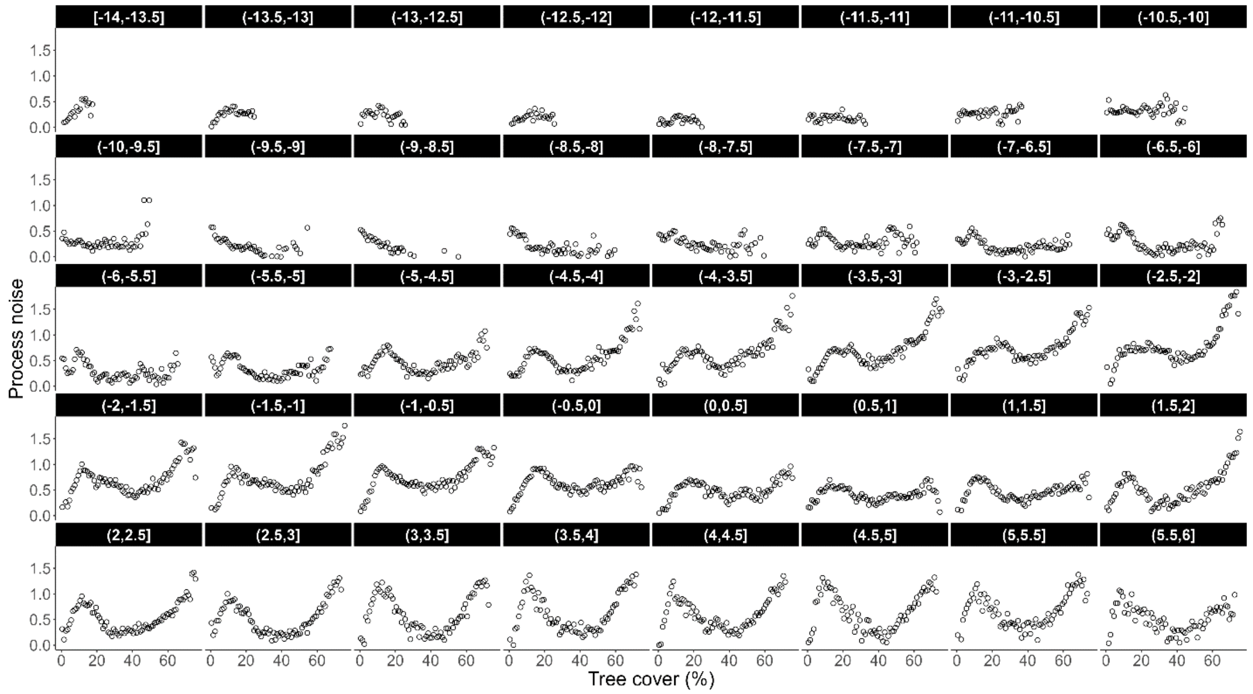


**Fig. S7. Tree cover-dependent process noise across temperature ranges.** Each panel represent a temperature range of 0.5°C between -14°C and 6°C.

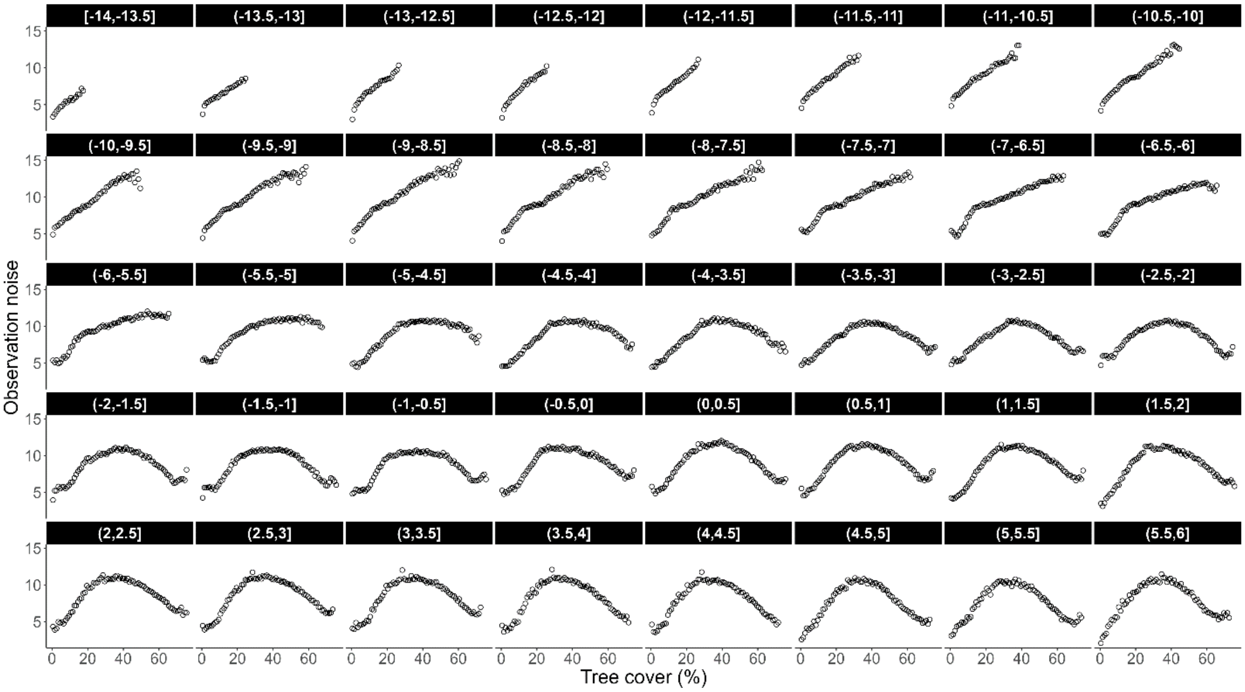


**Fig. S8. Tree cover-dependent observation noise across temperature ranges.** Each panel represent a temperature range of 0.5°C between -14°C and 6°C.

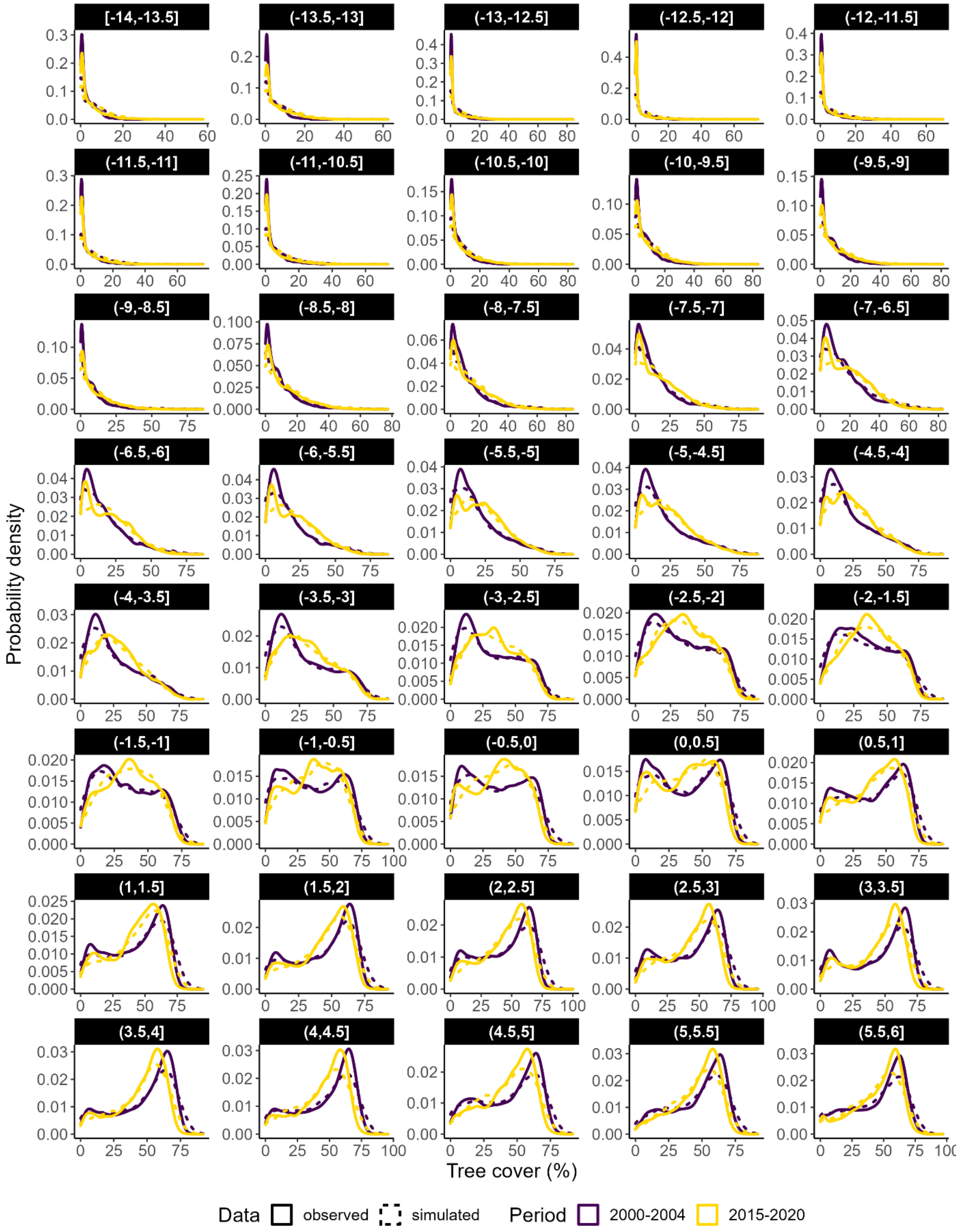


**Fig. S9. Comparison of observed and simulated tree cover 2000-2020.** Observed and simulated tree cover are shown for two time periods 2000-2004 and 2015-2020 across temperature ranges.

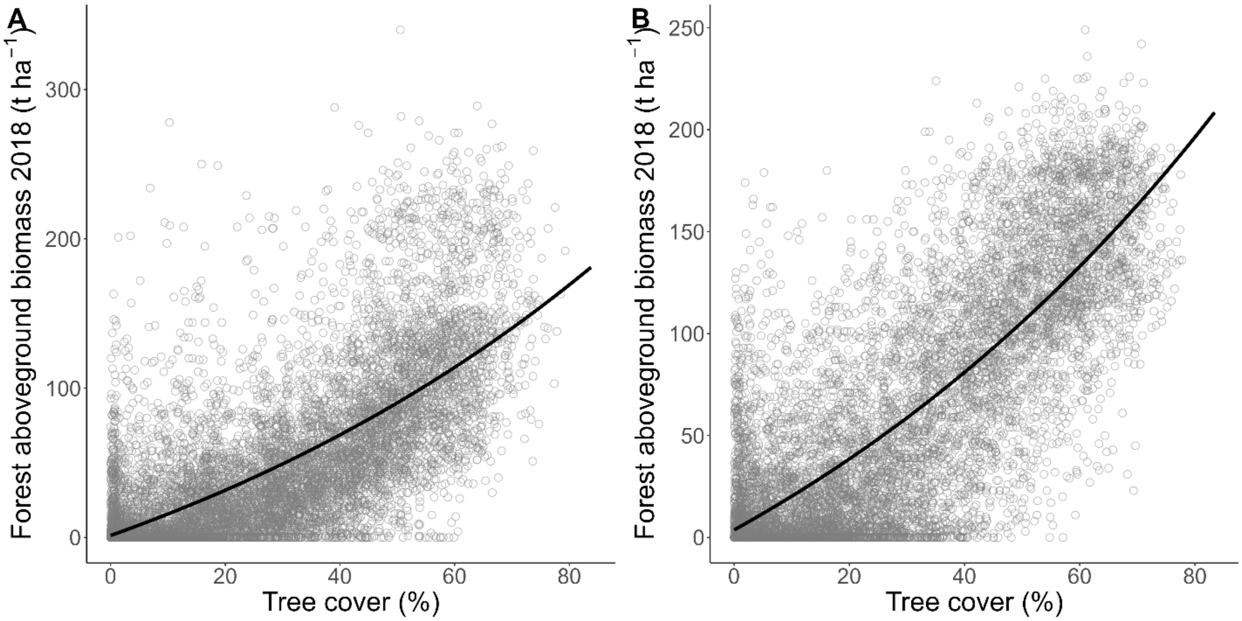


**Fig. S10. Relationship between tree cover and forest aboveground biomass.** Relationships are shown for **A** North America and **B** Eurasia for the year 2018. The solid line represents the fit of an exponential function (details are shown in Table S2). Grey data points are a random subset of 10,000 data points for better visualisation.

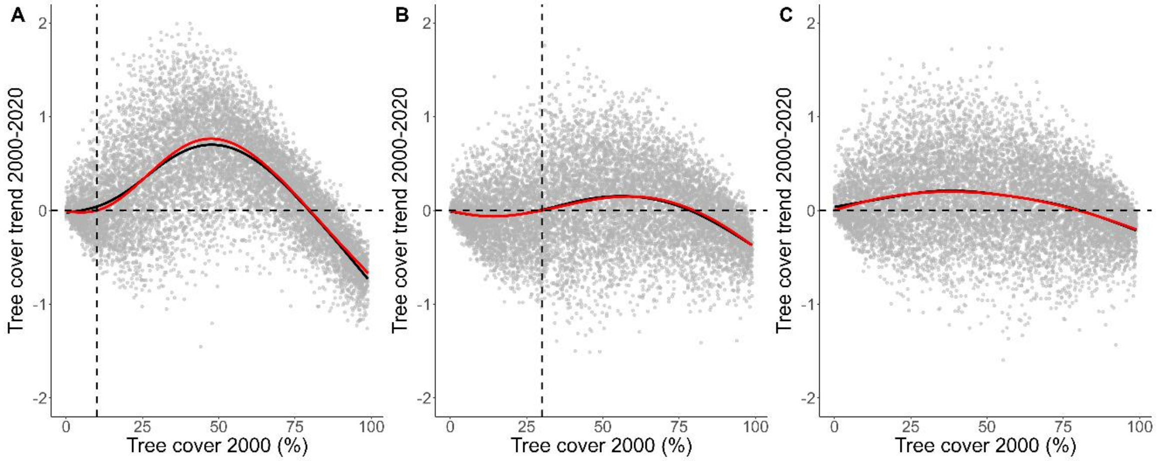


**Fig. S11. Validation of tree cover trend models through known models.** Tree cover time series were created using **A** an Allee effect model with a threshold of 10%, **B** an Allee effect model with a threshold of 30% and **C** a logistic growth model without Allee effect. Tree cover was simulated over 21 years with stochastic noise (process noise = 0.04, observation noise = 1). Grey points represent simulated data points from the models. Red lines represent the models without noise, black lines are model fits based on our approach.

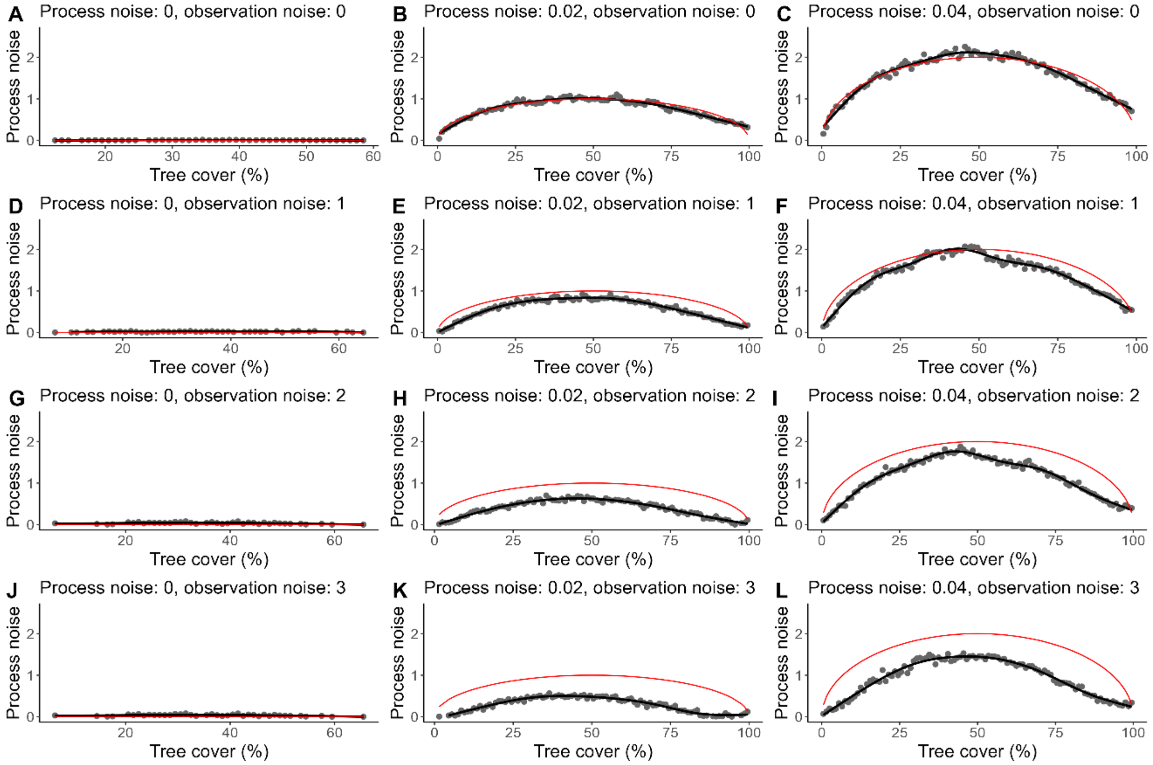


**Fig. S12. Estimated process and observation noise across tree cover gradient from Allee-effect models.** Tree cover was created through an Allee-effect model based on Equation S6 with C = 30%, K = 80%, process noise = 3 and observation noise = {0,1,2,3}. A-D Estimated process noise fitted through Equation S4 (points) with varying model observation noise. E-H Estimated observation noise across varying model observation noise. The red line indicates the true process or observation noise in the model. Black lines are generalized additive model (GAM) fits.

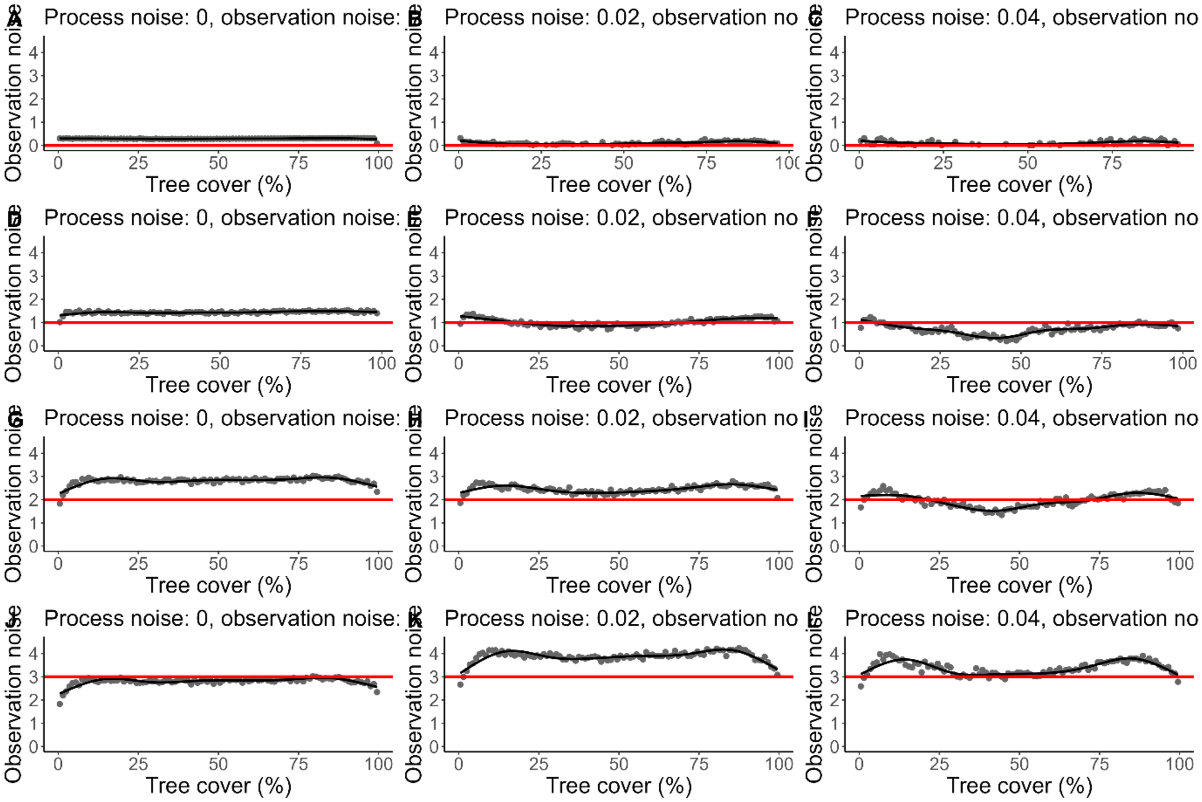


**Fig. S13. Estimated observation noise across tree cover gradient from Allee-effect models.** Tree cover was created through an Allee-effect model based on Equation S6 with C = 30%, K = 80%, process noise = {0, 0.02, 0.04} and observation noise = {0,1,2,3}. The panels along columns represent increasing process noise, along rows increasing observation noise. The red line indicates the true process or observation noise in the model. Black lines are GAM fits.

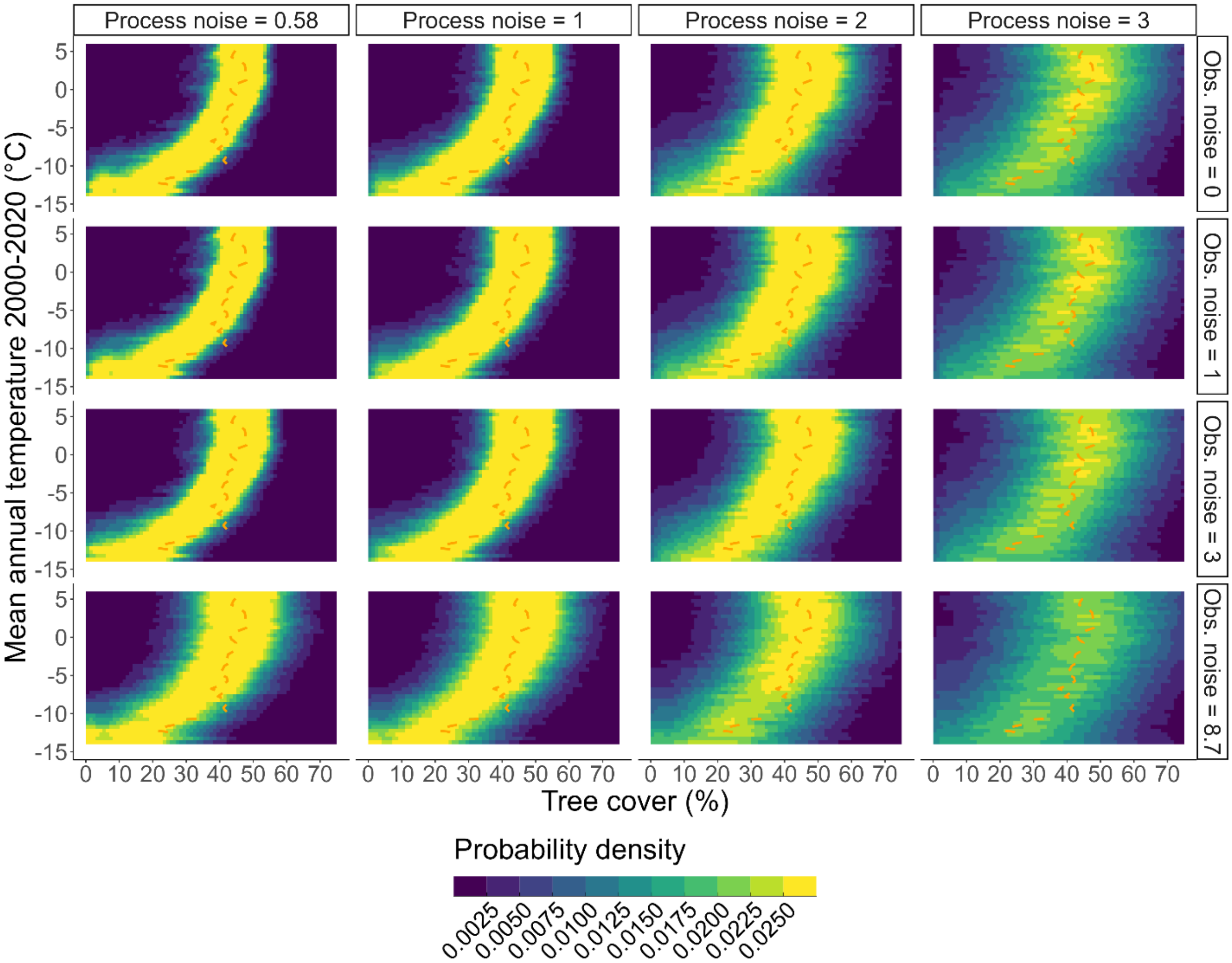


**Fig. S14. Projected probability densities of tree cover in the year 2100 for combinations of different process and observation noise.** Probability densities are shown across temperature gradients. The orange dashed line is the line of zero tree cover change around which tree cover distributions are expected. Process noise of 0.58 and observation noise of 8.7 are the measured median noise in the observed MODIS tree cover data. Model fitting and simulations were applied to all observed data across the global boreal biome.

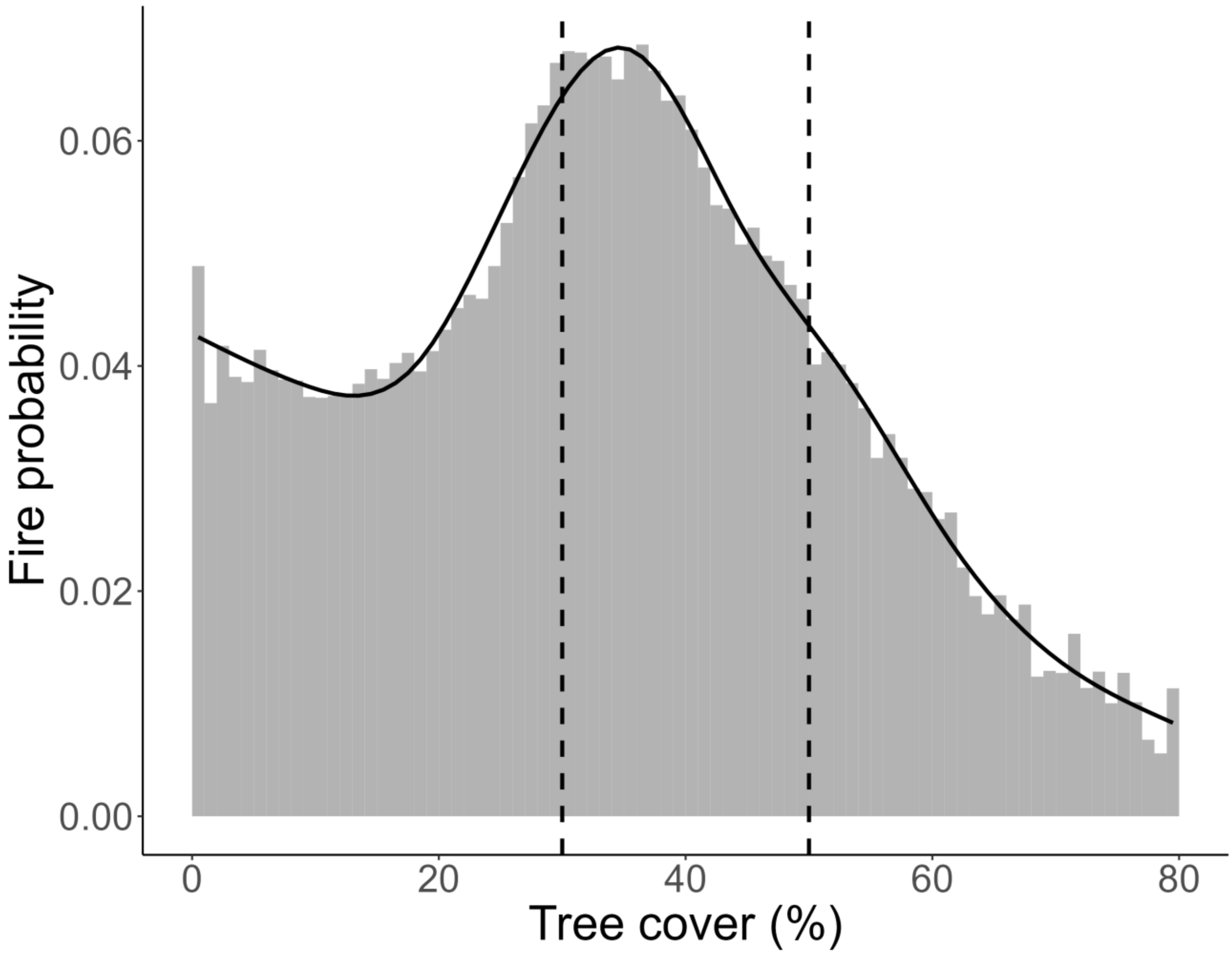


**Fig. S15. Fire probability across the boreal biome.** Fire probability is expressed as relative burnt area along the tree cover gradient. Dashed lines represent the expected range of tree cover by 2100.

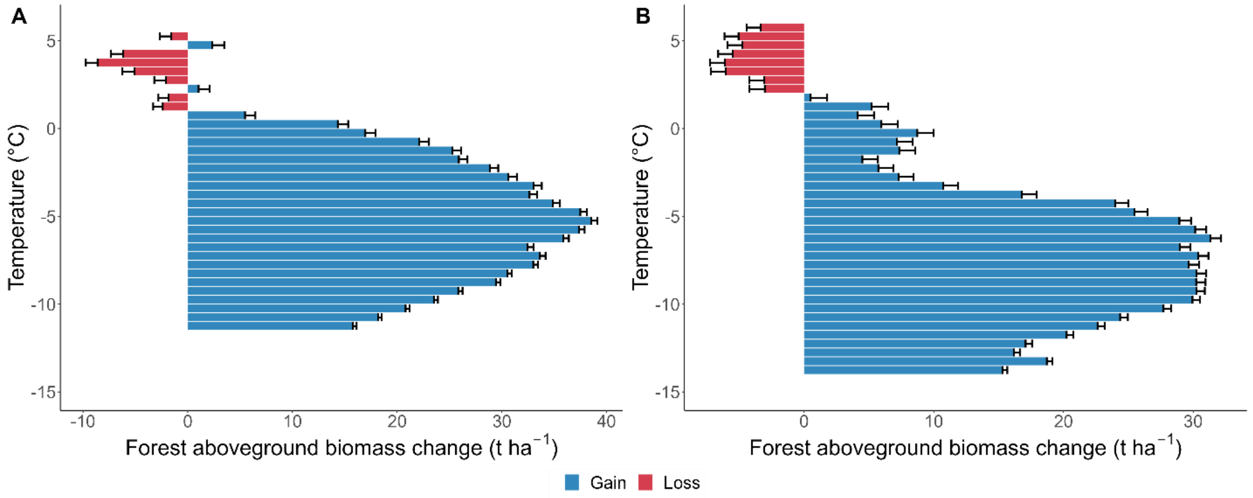


**Fig. S16. Relative forest aboveground biomass change between 2000 and 2100.** Biomass changes are shown for **A** North America and **B** Eurasia. Each bar represents the biomass change per temperature range. Error bars represent standard errors.

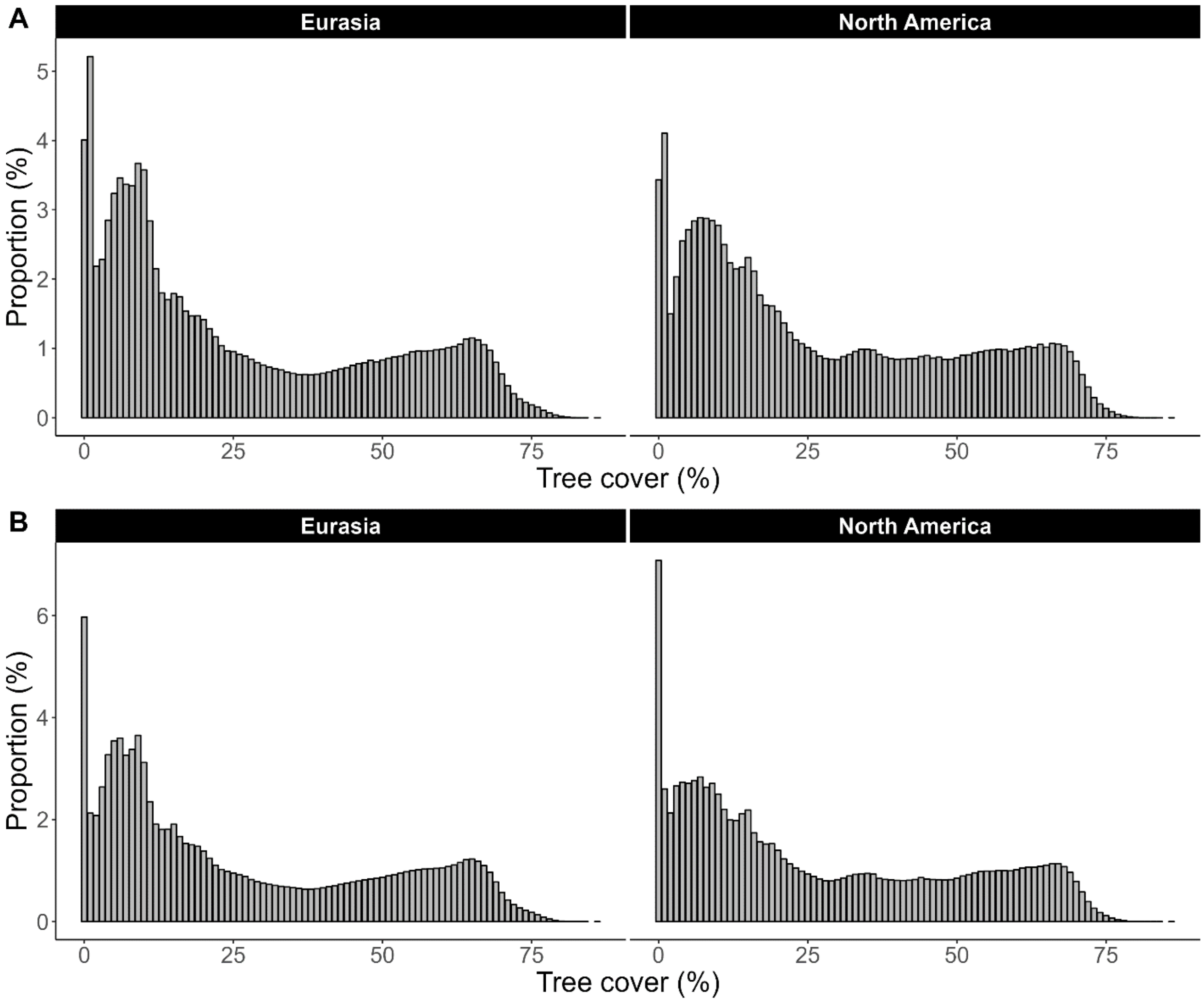


**Fig. S17. Representativeness of tree cover distributions within sample points compared to total boreal biome.** Distributions of tree cover are expressed as relative area proportions for Eurasia and North America within **A** sample points and **B** the entire boreal biome.

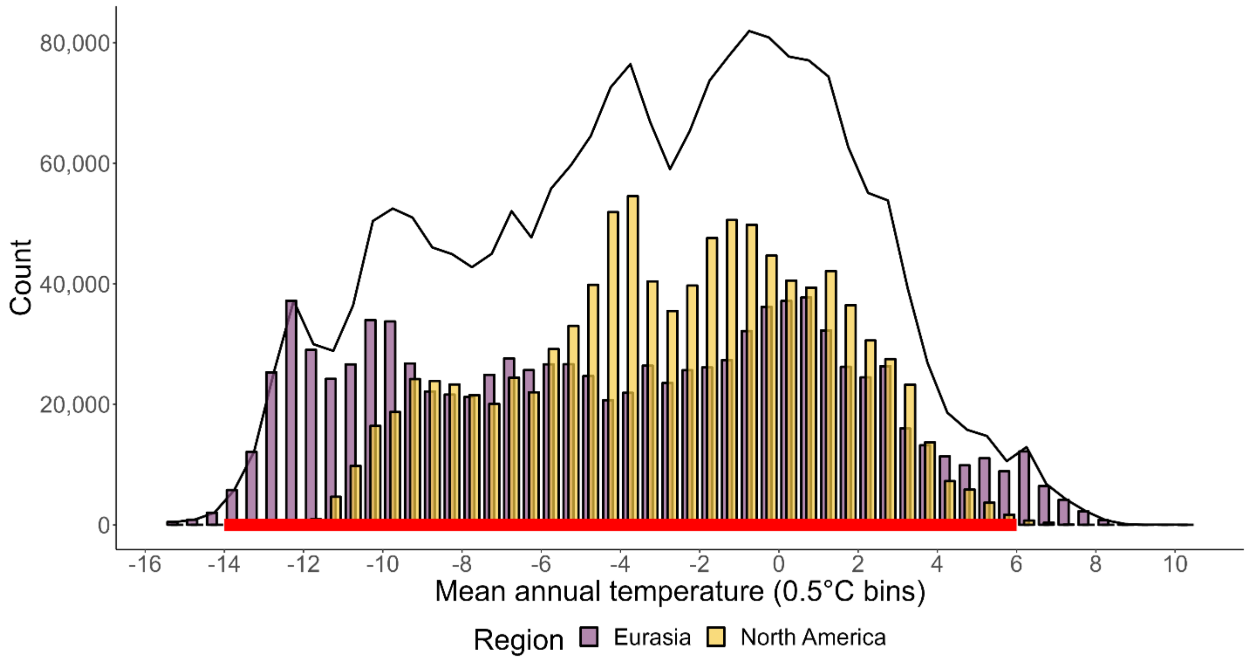


**Fig. S18. Frequency distributions of mean annual temperatures within global boreal forests.** Temperatures are separated in bins of 0.5°C and are shown for each continent (bars) and for the entire biome (black line). The red horizontal line represents the temperature range used in this study.

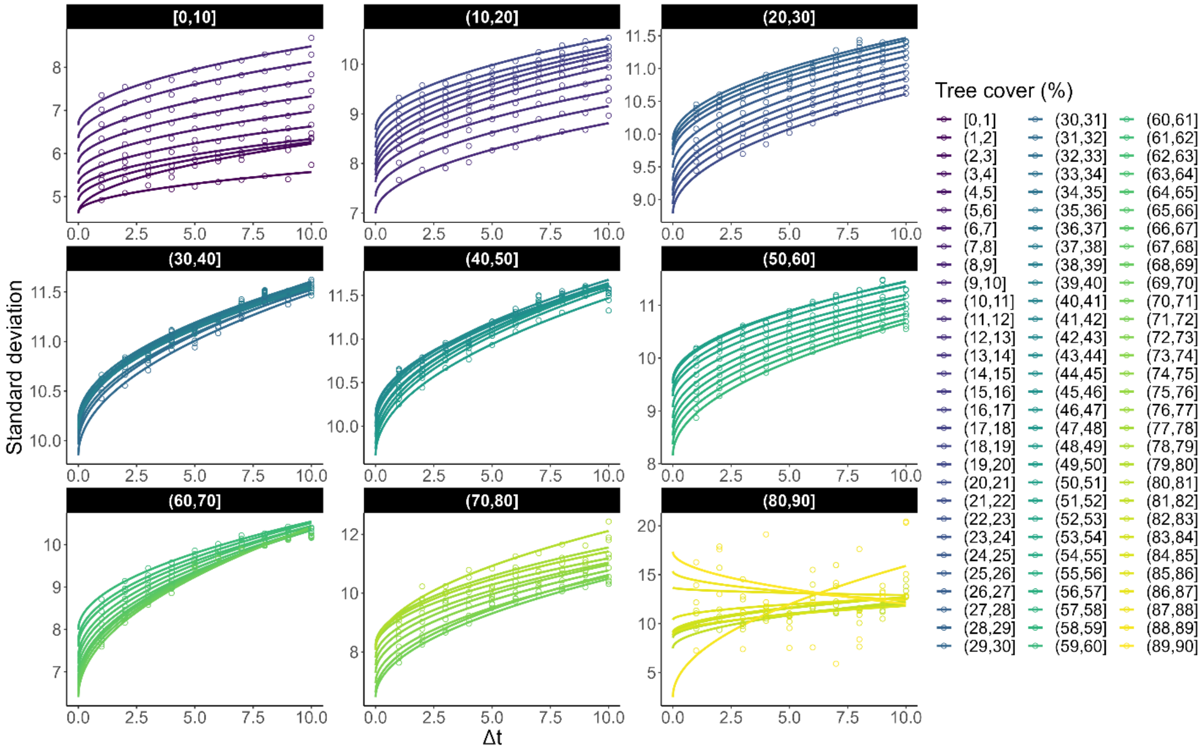


**Fig. S19. Standard deviations of tree cover with increasing time steps.** Colours represent groups of initial tree cover values. Points are standard deviations of tree cover for each group for the following 1-10 years (Δt). Lines are model fits based on the square root of Δt. Panels separate tree cover groups into groups of 10% tree cover.

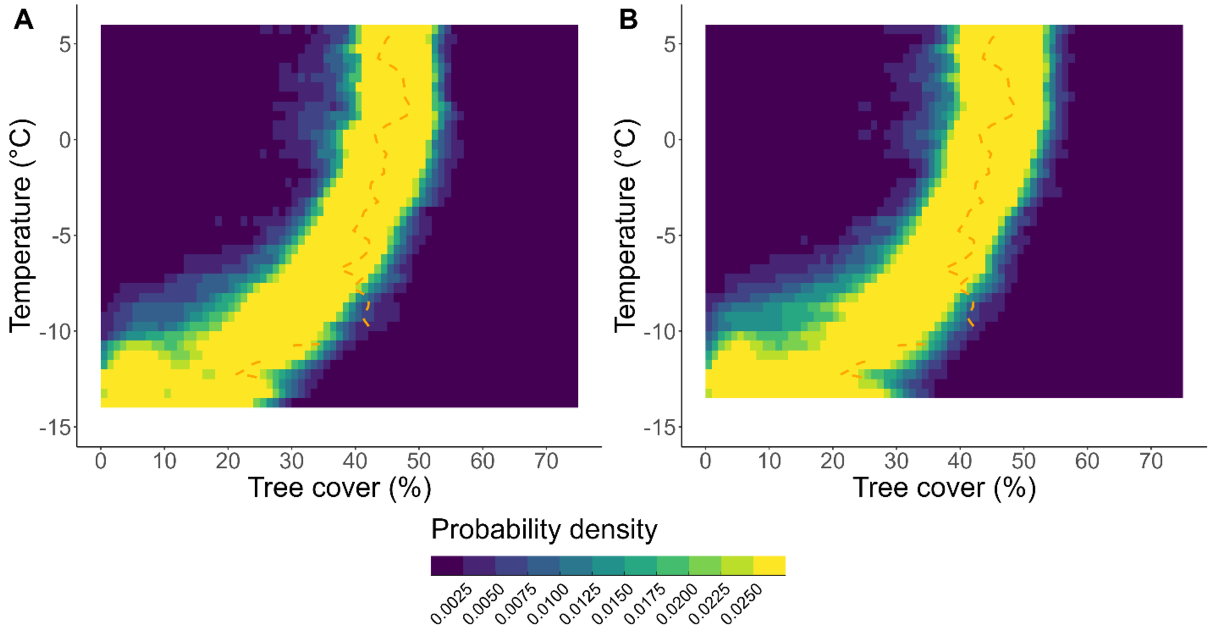


**Fig. S20. Comparison of expected tree cover distributions in 2100 for different assumptions of model noise. A** The expected tree cover where noise is fitted on different temperature bins. **B** The expected tree cover where we used a general noise function, fitted on all data points. Tree cover was simulated from the initial values in 2000 using tree cover changes 2000-2020. The dashed line is the line of zero change around which tree cover distributions are expected. Probability densities in both panels do not include observation noise.

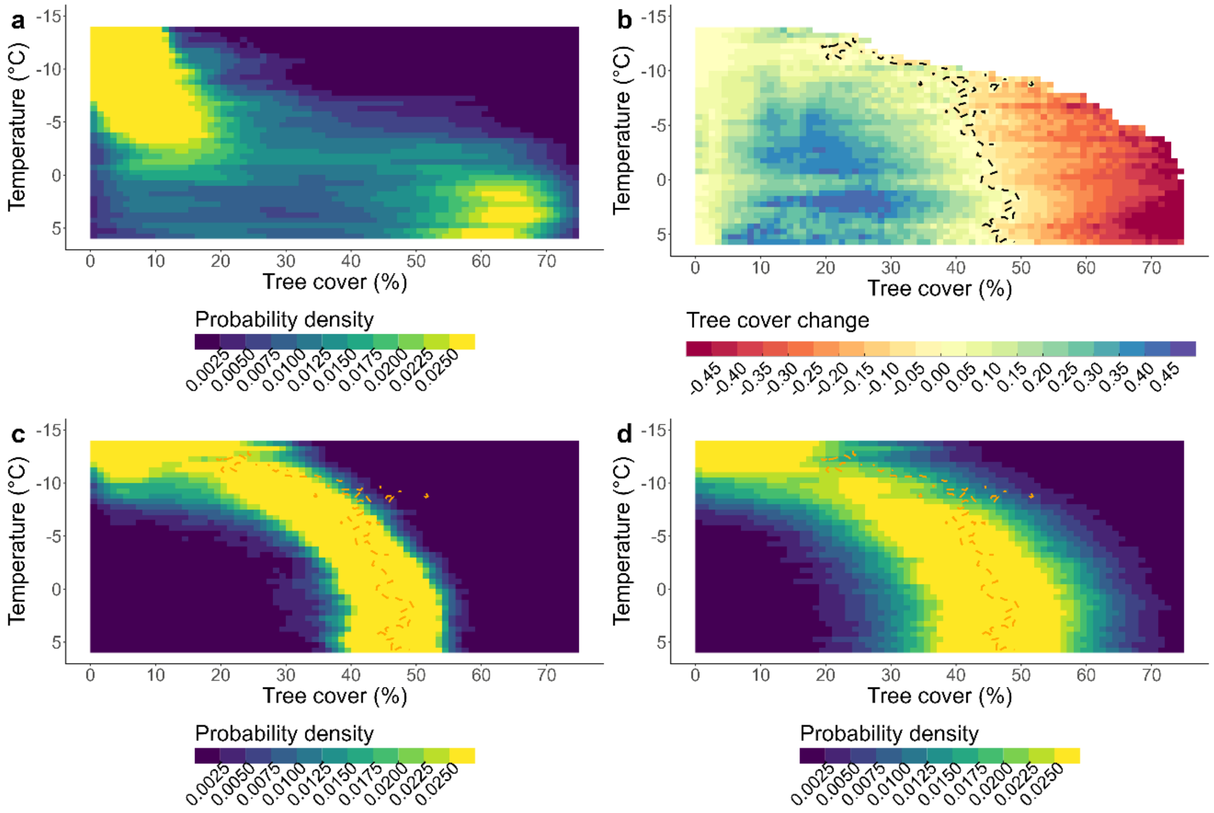


**Fig. S21. Tree cover distribution and change in global boreal forests using a binning approach in tree cover models.** Each panel represents distributions or change within a tree cover-temperature space. Tree cover is shown in ranges of 1%. Temperatures are mean annual temperatures 2000-2020 and are shown in ranges of 0.5°C. **A** Observed probability densities of tree cover in the year 2000. **B** Tree cover change between 2000 and 2020. The dashed line marks zero change and thus indicates potential tree cover states. **C** Expected probability densities of tree cover in the year 2100. Tree cover was simulated from the initial values in 2000 using tree cover changes 2000-2020. The dashed line is the line of zero change around which tree cover distributions are expected. **D** Expected probability densities of tree cover in the year 2100 including observation noise. Compare with model results using fitted models (Figure 2 in main text) instead of bins.

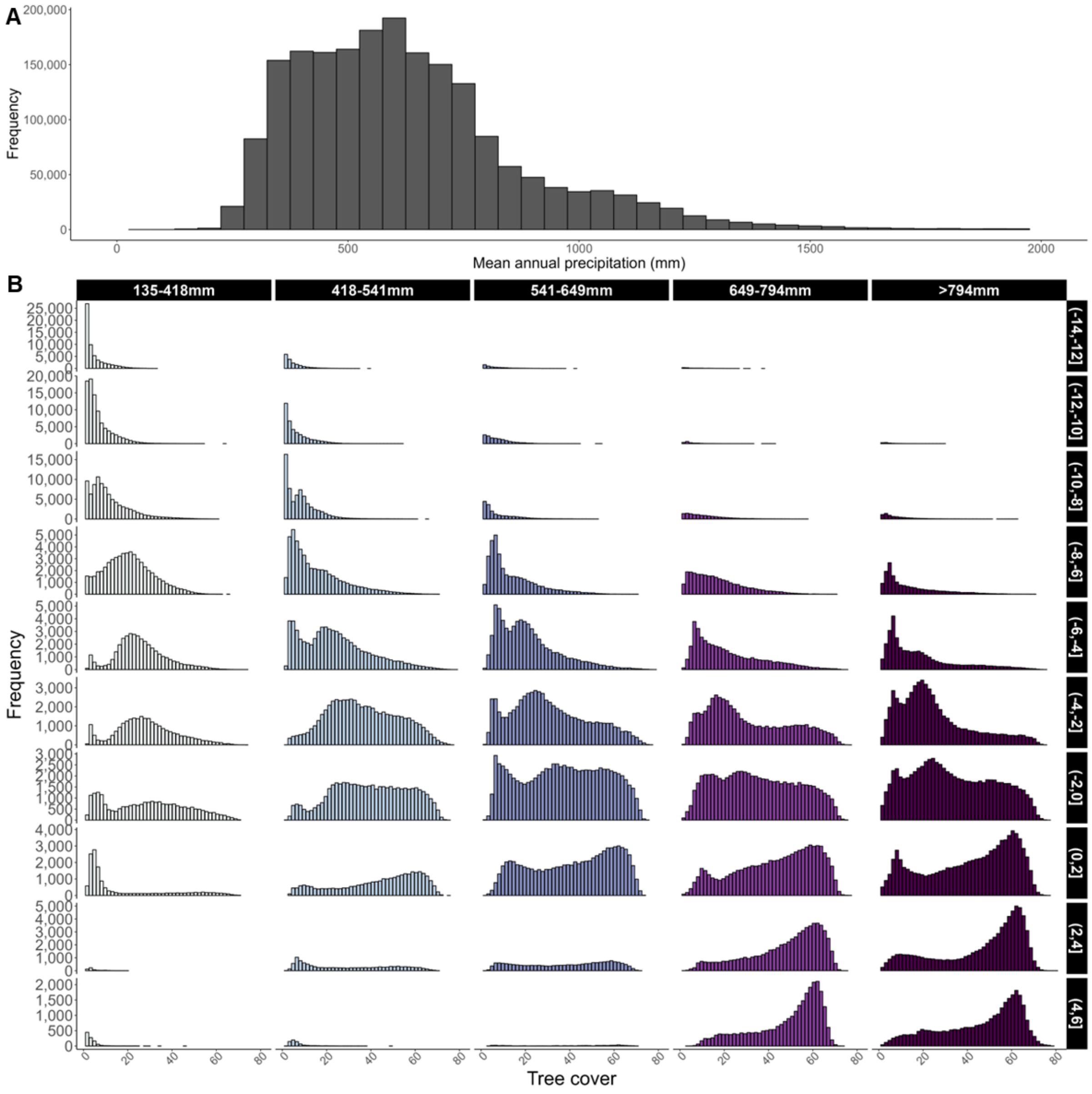


**Fig. S22. Tree cover distributions across the precipitation-temperature spectrum of the global boreal biome. A** Frequency distribution of mean annual precipitation 2000-2020 of 2,000,000 sample plots across the biome. **B** Frequency distribution of tree cover within temperature bins along rows (of 1 ˚C each) and precipitation bins along columns (of 20% quantiles).

## Table S1. Details on datasets used in this study.

| Name | Source | Time period | Temporal resolution | Spatial resolution | Pre-processing |
|---|---|---|---|---|---|
| Boreal forest boundary | Delineation based on Gauthier *et al.* 2015(2) | na | na | na | No pre-processing |
| Tree cover time series | MODIS Vegetation Continuous Field Collection 6(3) | 2000-2020 | annual | 250m | No pre-processing |
| Land cover classes | Copernicus Global Land Cover Map(4) | 2015 | na | 100m | We matched the spatial resolution with that of tree cover data (250 m) by resampling using the nearest neighbour method in ArcPro, version 2.8.3. |
| Climate data - Mean annual temperature and mean annual precipitation | European Centre for Medium-Range Weather Forecast re-analysis (ERA5)(5) | 2000-2020 | monthly | 0.25 decimal degrees (~21 km) | We extracted monthly surface temperatures and monthly precipitation expressed as average daily precipitation between 2000 and 2020. We converted temperatures into °C and calculated the mean annual temperature over the entire period 2000-2020. We converted precipitation to total monthly precipitation using the daily value provided. We then summed the monthly precipitation across each year and calculated the mean annual precipitation over all years 2000-2020. |
| Predicted mean annual temperatures | EC-Earth3-Veg-LR model(6) | 2020-2100 | monthly | 1.25 decimal degrees (~110km) | We extracted temperature predictions for scenario SSP3-7.0 as an intermediate carbon mitigation scenario. We calculated mean annual temperatures in °C for each year 2020-2100. |
| Forest fires | MODIS Burned Area(7) | 2000-2020 | irregular | 250m | We merged all available data within our study period to map the locations and years of each detected fire. |
| Forest management | Global forest management dataset version 3(8) | 2015 | na | 100m | The map depicts categories of managed and unmanaged forest areas. We matched the spatial resolution with that of tree cover data (250 m) by resampling using the nearest neighbour method in ArcPro, version 2.8.3. We then reclassified areas as 'Unmanaged' which did not have a management category or were classified as '11 - Naturally regenerating forest without any signs of human activities'. All other forest management classes were defined as 'Managed'. |
| Forest aboveground biomass | European Space Agency Biomass Climate Change Initiative version 3(9) | 2010, 2018 | na | 250m | We matched the spatial resolution with that of tree cover data (250 m) by resampling using the nearest neighbour method in ArcPro, version 2.8.3. |

**Table S2. Representativeness of sample points for total boreal biome.** Proportions of burnt and unburnt areas, as well as forest management classifications are compared for North America and Eurasia between sample points and the total boreal biome.

| Region | Management | Samples | Biome | Samples | Biome |
|---|---|---|---|---|---|
| | | Area (ha) | | Proportion (%) | |
| | Fire | | | | |
| Eurasia | Unburnt | 5,768,931 | 1,420,562,537 | 92.3 | 91.5 |
| Eurasia | Burnt | 481,069 | 132,100,313 | 7.7 | 8.5 |
| North America | Unburnt | 5,886,425 | 641,091,982 | 94.2 | 94.4 |
| North America | Burnt | 363,575 | 38,221,306 | 5.8 | 5.6 |
| | Forest Management | | | | |
| Eurasia | Unmanaged - Unclassified | 1,968,256 | 457,772,471 | 31.5 | 29.5 |
| Eurasia | Unmanaged - Naturally regenerating | 3,079,269 | 763,896,796 | 49.3 | 49.2 |
| Eurasia | Managed - Naturally regenerating | 1,059,363 | 290,014,477 | 16.9 | 18.7 |
| Eurasia | Managed - Planted forests | 140,288 | 40,123,511 | 2.2 | 2.6 |
| Eurasia | Managed - Agroforestry | 2,825 | 855,594 | 0.0 | 0.1 |
| North America | Unmanaged - Unclassified | 2,207,438 | 223,441,395 | 35.3 | 32.9 |
| North America | Unmanaged - Naturally regenerating | 3,384,044 | 373,586,624 | 54.1 | 55.0 |
| North America | Managed - Naturally regenerating | 650,356 | 81,297,165 | 10.4 | 12.0 |
| North America | Managed - Planted forests | 8,150 | 987,047 | 0.1 | 0.1 |
| North America | Managed - Agroforestry | 13 | 1,059 | 0.0 | 0.0 |

**Table S3. Biomass models based on tree cover in North America and Eurasia.** Model fit is based on an exponential function between tree cover and biomass of the year 2010 and 2018.

| North America | Estimate | SE | t | p |
|---|---|---|---|---|
| Intercept | -117.1 | 0.19 | -615 | 2.00E-16 |
| exp(Tree cover 2010/100) | 123.5 | 0.14 | 896 | 2.00E-16 |
| adjusted $R^2$ | 0.45 | | | |
| | | | | |
| Eurasia | | | | |
| Intercept | -141.8 | 0.16 | -878 | 2.00E-16 |
| exp(Tree cover 2010/100) | 154.3 | 0.12 | 1268 | 2.00E-16 |
| adjusted $R^2$ | 0.62 | | | |

| North America | Estimate | SE | t | p |
|---|---|---|---|---|
| Intercept | -135.6 | 0.19 | -725 | 2.00E-16 |
| exp(Tree cover 2018/100) | 137.0 | 0.13 | 1017 | 2.00E-16 |
| adjusted $R^2$ | 0.51 | | | |
| | | | | |
| Eurasia | | | | |
| Intercept | -153.4 | 0.16 | -958 | 2.00E-16 |
| exp(Tree cover 2018/100) | 157.1 | 0.12 | 1352 | 2.00E-16 |
| adjusted $R^2$ | 0.65 | | | |

## SI References


1. F. Courchamp, T. Clutton-Brock, B. Grenfell, Inverse density dependence and the Allee effect. Trends Ecol Evol 14, 405–410 (1999).
2. S. Gauthier, P. Bernier, T. Kuuluvainen, A. Z. Shvidenko, D. G. Schepaschenko, Boreal forest health and global change. Science (1979) 349, 819–822 (2015).
3. C. DiMiceli, et al., MOD44B MODIS/Terra Vegetation Continuous Fields Yearly L3 Global 250m SIN Grid V006. (2015).
4. M. Buchhorn, et al., Copernicus Global Land Service: Land Cover 100m: epoch 2015: Globe (Version V2.0.2 (2019). Available at: http://doi.org/10.5281/zenodo.3243509.
5. H. Hersbach, et al., ERA5 monthly averaged data on single levels from 1979 to present. (2019).
6. C. D. S. Copernicus Climate Change Service, CMIP6 climate projections. (2021).
7. L. Giglio, L. Boschetti, D. P. Roy, M. L. Humber, C. O. Justice, The Collection 6 MODIS burned area mapping algorithm and product. Remote Sens Environ 217, 72–85 (2018).
8. M. Lesiv, et al., Global forest management data for 2015 at a 100 m resolution. Sci Data 9 (2022).
9. M. Santoro, O. Cartus, ESA Biomass Climate Change Initiative (Biomass_cci): Global datasets of forest above-ground biomass for the years 2010, 2017 and 2018, v3. (2021).

**Software S1 (separate file).** R-code used for modeling.
